# Synthesis of a Side Chain Alkyne Analogue of Sitosterol as a Chemical Probe for Imaging in Plant Cells

**DOI:** 10.3390/biom14050542

**Published:** 2024-04-30

**Authors:** Miriam Hollweck, David Jordan, Franz Bracher

**Affiliations:** Department of Pharmacy, Ludwig-Maximilians University Munich, Butenandtstraße 5–13, 81377 Munich, Germany; miriam.hollweck@cup.lmu.de (M.H.); david.jordan99@t-online.de (D.J.)

**Keywords:** phytosterols, sitosterol, click chemistry, imaging, Johnson–Claisen rearrangement, alkyne

## Abstract

Clickable chemical tools are essential for studying the localization and role of biomolecules in living cells. For this purpose, alkyne-based close analogs of the respective biomolecules are of outstanding interest. Here, in the field of phytosterols, we present the first alkyne derivative of sitosterol, which fulfills the crucial requirements for such a chemical tool as follows: very similar in size and lipophilicity to the plant phytosterols, and correct absolute configuration at C-24. The alkyne sitosterol FB-DJ-1 was synthesized, starting from stigmasterol, which comprised nine steps, utilizing a novel alkyne activation method, a Johnson–Claisen rearrangement for the stereoselective construction of a branched sterol side chain, and a Bestmann–Ohira reaction for the generation of the alkyne moiety.

## 1. Introduction

Steroidal compounds are widespread in higher living organisms, as exemplified by cholesterol, as well as metabolically derived steroid hormones in eukariotic cells [1], ergosterol as an essential cell membrane component in fungi (and its biosynthesis as one of the major targets of antifungals [2]), and phytosterols in plants. Phytosterols exhibit numerous important functions in plant cells [3], and numerous beneficial pharmacological activities have been described for them [4].

Nevertheless, the biosynthesis, cellular localization, and metabolism of phytosterols is still the subject of many in-depth investigations. One important methodology in this field is the microscopic imaging of sterols in living cells. In the end, this approach uses fluorescent derivatives of the relevant sterols, obtained by the coupling of appropriately substituted steroidal chemical tools with tailored fluorescent dyes [5].

At present, the most important approach for imaging small molecules (physiological substances, natural products, drug candidates) in cells utilizes “click chemistry”. This chemical ligation technology was defined first by K. B. Sharpless in 2001 [6], and he was (together with C. Bertozzi and M. Meldal) awarded with the Nobel Prize in Chemistry “for the development of click chemistry and bioorthogonal chemistry” in 2022.

Click chemistry is among the bio-orthogonal methods [7], meaning chemical reactions that can occur inside of living organisms without (too much) interfering with the native biochemical processes [8]. Among the most important click reactions is the 1,3-dipolar cycloaddition between organic azides and alkynes, performed as copper-catalyzed azide-alkyne cycloaddition (CuAAC) [9], or as strain-promoted azide-alkyne cycloaddition (SPAAC) [10].

Click chemistry has meanwhile found ample applications in steroid chemistry, with applications in organic synthesis, material science, drug discovery, and bioconjugation chemistry [9,11]. Recently, CuAAC has also been combined with photo-crosslinking (“PhotoClick cholesterol”) for the identification of sterol-binding proteins in yeast cells by means of quantitative proteomics [12].

Clickable steroids for biochemical investigations have, up to now, been developed mainly for cholesterol; exceptions include a lanosterol side chain alkyne [13] and several side chain alkyne derivatives of cholesterol (**1**) biosynthesis intermediates (a-Chol, a-7-DHC, a-DHCEp) [14] from the Porter lab. Only one cholesterol side chain alkyne with a complete isooctyl side chain has been published [15]; complementary clickable cholesterol azides are only published with truncated side chains [16,17]. A couple of clickable steroids have been derived from cholesterol (**1**) and bile acids via the functionalization of the 3-hydroxy group by means of etherification or esterification with short-chain alkynes [18,19,20] or azides [19,20]. However, this modification suffers from the elimination of the physiologically important hydroxy group in the sterols, resulting in the significantly changed lipophilicity of the tools, and, in the case of esters, unpredictable hydrolytic stability in living cells. Based on such considerations, the Salic group attached an alkyne functionality at C-19 in cholesterol (**1**) [5].

**Figure 1:**
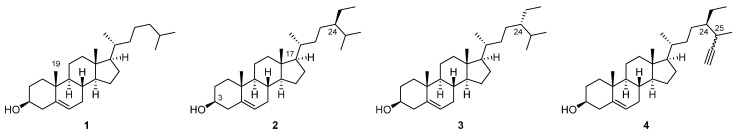
Structures of cholesterol (**1**), sitosterol (**2**), clionasterol (**3**), and the clickable target compound FB-DJ-1 (**4**).

In contrast to cholesterol (**1**), phytosterols, like sitosterol (**2**) and stigmasterol, have a more complex side chain at C-17, with a typical ethyl branching, resulting in a 24α-configured additional stereocenter. Sterols with 24β-configuration, like clionasterol (**3**) and poriferasterol, are rather found in algae [21] (Figure 1).

Until now, clickable sitosterol (**2**) derivatives have only been obtained via the etherification of 3-OH with a propargyl residue [22] and by the conversion of the 3β-configured secondary alcohol into a 3α-azide [23]. A related side chain azide has only been derived from diosgenin [24].

In the present project, we aimed at developing a clickable sitosterol alkyne (FB-DJ-1, **4**) with the alkyne group at the terminus of the side chain, and with the correct 24α configuration (Figure 1). By converting one of the two methyl groups of the terminal isopropyl moiety into an ethynyl unit, neither the size nor lipophilicity of the original phytosterol should be drastically altered, so we expect that this new chemical tool should perfectly mimic sitosterol in its biological behavior, especially distribution in living organisms. This modification will, however, generate a new stereocenter at C-25. Since which configuration at C-25 would be advantageous in cells was unpredictable, we decided to synthesize the target sitosterol alkyne FB-DJ-1 (**4**) as an epimeric mixture with 24*R*,25*RS* configuration.

## 2. Materials and Methods

^1^H and ^13^C NMR spectra were recorded with either Avance III HD 400 MHz Bruker BioSpin or Avance III HD 500 MHz Bruker BioSpin spectrometers (Bruker Bio-Spin, Billerica, MA, USA). Chemical shifts are given in parts per million (ppm). *J* values are given in Hertz (Hz). Standard abbreviations are used to denote multiplicities. Signal assignments were carried out based on ^1^H, ^13^C, HMBC, HSQC, and COSY spectra. EI mass spectra were recorded on a Jeol Mstation 700 or JMS GCmate II Jeol (Jeol, Tokyo, Japan). Purification via flash column chromatography was performed using Silica Gel 60 (Merck, Darmstadt, Germany). All reactions were monitored with TLC using precoated plastic sheets POLYGRAM from Macherey-Nagel (Düren, Germany). Detection was either carried out using the CAM stain (ceric ammonium molybdate) followed by heating, or UV light at 254 nm. The melting points were determined using a Büchi Melting Point B-540 device (Büchi Labortechnik AG, Flawil, Switzerland). Optical rotation was measured using a 241 MC polarimeter from Perkin Elmer (Waltham, MA, USA). All measurements were carried at room temperature in CHCl_3_; the concentrations are given in g/100 mL. HPLC purities were determined using an Agilent G1311A QuatPump with a G1315C DAD SL detector (210 nm); column: Zorbax C18 SB (3.5 µm), eluent: methanol, phosphate buffer pH 5 (99:1), temperature 50 °C, flow rate: 1 mL/min. 

(22*R*)-6β-Methoxy-3α,5-cyclo-27-nor-5α-cholest-23-yn-22-ol (**9a**) and

(22*S*)-6β-Methoxy-3α,5-cyclo-27-nor-5α-cholest-23-yn-22-ol (**9b**) 

To a stirred solution of but-1-yn-1-yltrimethylsilane (**8**; 3.3 mL, 19 mmol) and TBAF (1 M in THF, 0.97 mL, 0.97 mmol) in dry THF (50 mL) under nitrogen atmosphere, a solution of (20*S*)-6β-methoxy-3α,5-cyclo-5α-pregnane-20-carboxaldehyde (**7**) (1.7 g, 4.8 mmol) in dry THF (40 mL) was added dropwise over the course of 30 min. The reaction mixture was stirred for another 15 min, then hydrochloric acid (1 M, 1.0 mL) was added and stirring continued for 15 min. The mixture was neutralized with an aqueous NaHCO_3_ solution, and the biphasic mixture was extracted with ethyl acetate (3 × 100 mL). The combined organic extracts were dried using a phase separation paper, and the solvent was removed *in vacuo*. The crude product was subjected to column chromatography (isohexanes/ethyl acetate 95:5) to first provide (22*R*)-6β-methoxy-3α,5-cyclo-27-nor-5α-cholest-23-yn-22-ol (**9a**) (0.36 g, 0.91 mmol, 19%) as a colorless solid, and then (22*S*)-6β-methoxy-3α,5-cyclo-27-nor-5α-cholest-23-yn-22-ol (**9b**) (0.67 g, 1.7 mmol, 35%) as a colorless oil.

**9a:** ^1^H NMR (500 MHz, DMSO) *δ* 4.87 (d, *J* = 5.2 Hz, 1H, 22-OH), 4.37–4.30 (m, 1H, H-22), 3.21 (s, 3H, OCH_3_), 2.73 (t, *J* = 2.8 Hz, 1H, H-6), 2.17 (qd, *J* = 7.5, 1.9 Hz, 2H, C-25), 1.84–1.23 (m, 13H), 1.17–0.98 (m, 7H), 0.95 (s, 3H, H-19), 0.90 (d, *J* = 6.2 Hz, 3H, H-21), 0.87–0.75 (m, 3H, H-1, H-3 and H-9), 0.63 (s, 3H, H-18), 0.59 (t, *J* = 4.3 Hz, 1H, H-4), 0.40 (dd, *J* = 8.0, 4.9 Hz, 1H, H-4). ^13^C NMR (126 MHz, DMSO) *δ* 84.7 (C, C-23), 82.3 (C, C-24), 81.2 (CH, C-6), 62.9 (C, C-22), 55.9 (CH_3_, OCH_3_), 55.6 (CH, C-14), 50.4 (CH, C-17), 47.5 (CH, C-9), 43.0 (C, C-10), 42.2 (C, C-13), 40.3 (CH, C-20), 38.7 (CH_2_, C-12), 34.9 (C, C-5), 34.8 (CH_2_, C-7), 32.8 (CH_2_, C-1), 30.0 (CH, C-8), 26.3 (CH_2_, C-16), 24.6 (CH_2_, C-2), 23.8 (CH_2_, C-15), 22.2 (CH_2_, C-11), 20.8 (CH, C-3), 19.3 (CH_3_, C-19), 14.0 (CH_3_, C-26), 13.7 (CH_3_, C-21), 12.8 (CH_2_, C-4), 12.5 (CH_2_, C-25), 11.7 (CH_3_, C-18). HREIMS *m*/*z* 398.3179 (calcd for [C_27_H_42_O_2_]^•+^, 398.3179). m.p.: 133 °C, [α] = −9.5° (c = 0.11)

**9b:** ^1^H NMR (500 MHz, DMSO) *δ* 4.84 (d, *J* = 5.5 Hz, 1H, 22-OH), 4.24 (dq, *J* = 5.4, 1.9 Hz, 1H, H-22), 3.21 (s, 3H, OCH_3_), 2.75–2.71 (m, 1H, H-6), 2.17 (qd, *J* = 7.5, 1.9 Hz, 2H, C-25), 1.90 (dt, *J* = 12.5, 3.4 Hz, 1H, C-12), 1.84–1.22 (m, 12H), 1.13–0.98 (m, 10H), 0.95 (s, 3H, H-19), 0.89 (dd, *J* = 8.0, 4.0 Hz, 1H, H-3), 0.85–0.76 (m, 2H, H-1 and H-9), 0.65 (s, 3H, H-18), 0.59 (t, *J* = 4.3 Hz, 1H, H-4), 0.40 (dd, *J* = 7.9, 4.9 Hz, 1H, H-4). ^13^C NMR (126 MHz, DMSO) *δ* 84.7 (C, C-23), 82.4 (C, C-24), 81.2 (CH, C-6), 63.2 (C, C-22), 55.9 (CH_3_, OCH_3_), 55.8 (CH, C-14), 51.4 (CH, C-17), 47.2 (CH, C-9), 42.9 (C, C-10), 42.3 (C, C-13), 42.0 (CH, C-20), 39.3 (CH_2_, C-12, underneath the solvent signal), 34.9 (C, C-5), 34.8 (CH_2_, C-7), 32.8 (CH_2_, C-1), 30.1 (CH, C-8), 27.0 (CH_2_, C-16), 24.6 (CH_2_, C-2), 23.8 (CH_2_, C-15), 22.3 (CH_2_, C-11), 20.8 (CH, C-3), 19.3 (CH_3_, C-19), 14.0 (CH_3_, C-26), 13.5 (CH_3_, C-21), 12.8 (CH_2_, C-4), 12.0 (CH_2_, C-25), 11.7 (CH_3_, C-18). HREIMS *m*/*z* 398.3180 (calcd for [C_27_H_42_O_2_]^•+^, 398.3179).

(22*R*,23*Z*)-6β-Methoxy-3α,5-cyclo-27-nor-5α-cholest-23-en-22-ol (**10a**)

This compound was synthesized from alkynol **9a** (0.17 g, 0.43 mmol) via catalytic hydrogenation using Lindlar’s catalyst, according to procedure from the literature [25], providing **10a** as a colorless sticky oil (96 mg, 0.24 mmol, 56%). ^1^H NMR (500 MHz, CDCl_3_) *δ* 5.50–5.38 (m, 2H, H-23 and H-24), 4.69–4.64 (m, 1H, H-22), 3.33 (s, 3H, OCH_3_), 2.78 (t, *J* = 2.9 Hz, 1H, H-6), 2.19–2.00 (m, 2H, H-25), 1.94 (ddt, *J* = 37.0, 13.6, 3.3 Hz, 2H, H-1 and H-12), 1.80–1.57 (m, 4H), 1.54–1.23 (m, 8H), 1.17–1.06 (m, 3H), 1.03 (s, 3H, H-19), 1.00 (t, *J* = 7.5 Hz, 3H, H-26), 0.90–0.83 (m, 7H), 0.76 (s, 3H, H-18), 0.65 (dd, *J* = 5.1, 3.8 Hz, 1H, H-4), 0.44 (dd, *J* = 8.0, 5.1 Hz, 1H, H-4). ^13^C NMR (126 MHz, CDCl_3_) *δ* 133.1 (CH, C-23 or C-24), 131. (CH, C-23 or C-24)C, 82.6 (CH, C-6), 69.5 (CH, C-22), 56.8 (CH_3_, OCH_3_), 56.4 (CH, C-14), 51.9 (CH, C-17), 48.3 (CH, C-9), 43.6 (C, C-10), 42.9 (C, C-13), 40.7 (CH, C-20), 39.8 (CH_2_, C-12), 35.4 (C, C-5), 35.2 (CH_2_, C-1), 33.5 (CH_2_, C-7), 30.6 (CH, C-8), 27.2 (CH_2_, C-16), 25.1 (CH_2_, C-2), 24.2 (CH_2_, C-15), 22.9 (CH_2_, C-11), 21.6 (CH, C-3), 21.5 (CH_2_, C-25), 19.4 (CH_3_, C-19), 14.5 (CH_3_, C-26), 13.2 (CH_2_, C-4), 13.1 (CH_3_, C-18), 12.5 (CH_3_, C-21). HREIMS *m*/*z* 400.3330 (calcd for [C_27_H_44_O_2_]^•+^, 400.3336).

(22*R*,23*E*)-6β-Methoxy-3α,5-cyclo-27-nor-5α-cholest-23-en-22-ol (**10b**)

In a flame-dried Schlenk flask, LiAlH_4_ (0.51 g, 13 mmol) was suspended in dry THF (30 mL) under a nitrogen atmosphere. A solution of (22*S*)-6β-methoxy-3α,5-cyclo-27-nor-5α-cholest-23-yn-22-ol (**9b**) (0.67 g, 1.7 mmol) in dry THF (25 mL) was added, and the mixture was stirred overnight under reflux. After cooling to room temperature, the reaction was quenched by the addition of hydrochloric acid (1 M, 20 mL) and brine (20 mL), then was extracted with DCM (3 × 50 mL). The combined organic extracts were dried with a phase separation paper, and the solvent was removed *in vacuo*. Purification via column chromatography (isohexanes/ethyl acetate 90:10) provided (22*R*,23*E*)-6β-methoxy-3α,5-cyclo-27-nor-5α-cholest-23-en-22-ol (**10b**) (0.48 g, 1.2 mmol, 72%) as a colorless solid. ^1^H NMR (400 MHz, CDCl_3_) *δ* 5.66 (dtd, *J* = 15.5, 6.2, 1.4 Hz, 1H, H-24), 5.49 (ddt, *J* = 15.5, 5.3, 1.5 Hz, 1H, H-2′), 4.21 (t, *J* = 5.4 Hz, 1H, H-22), 3.33 (s, 3H, OCH_3_), 2.77 (t, *J* = 2.9 Hz, 1H, H-6), 2.12–2.02 (m, 2H, H-4′), 1.93 (ddt, *J* = 26.2, 13.6, 3.3 Hz, 3H, H-12 and CH_2_), 1.83–1.59 (m, 3H, H-8 and CH_2_), 1.55–1.30 (m, 7H), 1.21–0.98 (m, 10H), 0.93–0.78 (m, 6H, H-3, H-9, H-21 and CH_2_), 0.73 (s, 3H, H-18), 0.67–0.62 (m, 1H, H-4), 0.43 (dd, *J* = 8.0, 5.1 Hz, 1H, H-4). ^13^C NMR (101 MHz, CDCl_3_) *δ* 132.5 (CH, C-24), 131.7 (CH, C-23), 82.6 (CH, C-6), 74.3 (CH, C-22), 56.7 (CH_3_, OCH_3_), 56.5 (CH, C-14), 52.8 (CH, C-17), 48.1 (CH, C-9), 43.5 (C, C-10), 42.9 (CH_2_,C-13), 41.6 (CH, C-20), 40.4 (CH_2_, C-12), 35.4 (C, C-5), 35.2 (CH_2_), 33.5 (CH_2_), 30.7 (CH, C-8), 28.0 (CH_2_), 25.5 (CH_2_), 25.1 (CH_2_, C-25), 24.4 (CH_2_), 22.9 (CH_2_), 21.7 (CH, C-3), 19.4 (CH_3_, C-19), 13.8 (CH_3_, C-26), 13.2 (CH_2_, C-4), 12.4 (CH_3_, C-21), 12.1 (CH_3_, C-18). HREIMS *m*/*z* 400.3331 (calcd for [C_27_H_44_O_2_]^•+^, 400.3336). m.p.: 135 °C. [α] = 26.6° (c = 0.23).

Ethyl (22*E*,24*R*,25*RS*)-6β-Methoxy-3α,5-cyclo-5α-stigmast-22-en-26-oate (**11**)

To a solution of (22*R*,23*E*)-6β-ethoxy-3α,5-cyclo-27-nor-5α-cholest-23-en-22-ol (**10b**) (0.48 g, 1.2 mmol) in xylene (45 mL), triethyl orthopropionate (3.8 mL, 19 mmol) and propionic acid (89 µL, 1.2 mmol) were added under a nitrogen atmosphere, and the mixture was heated to reflux for 1.25 h. After cooling to room temperature, saturated Na_2_CO_3_ solution (50 mL) was added, and the biphasic mixture was extracted with ethyl acetate (3 × 50 mL). The combined organic extracts were washed with water (2 × 100 mL) and brine (100 mL), and then dried using a phase separation paper. After the solvent had been removed *in vacuo*, the crude product was subjected to column chromatography (isohexanes/ethyl acetate 95:5) to provide ethyl (22*E*,24*R*,25*RS*)-6β-methoxy-3α,5-cyclo-5α-stigmast-22-en-26-oate (**11**) (0.45 g, 0.93 mmol, 78%) as a colorless oil. ^1^H NMR (400 MHz, CDCl_3_) *δ* 5.29–5.19 (m, 1H, H-22), 5.08 (dd, *J* = 15.1, 9.2 Hz, 0.46H, H-23), 4.95 (dd, *J* = 15.1, 9.4 Hz, 0.61H, H-23), 4.17–4.03 (m, 2H, H-1′), 3.32 (d, *J* = 0.7 Hz, 3H, OCH_3_), 2.77 (t, *J* = 2.4 Hz, 1H, H-6), 2.40–2.28 (m, 1H, H-2′), 2.14–1.57 (m, 8H), 1.53–0.96 (m, 24H), 0.90–0.79 (m, 6H, H-1, H-3, H-9 and H-29), 0.72 (d, *J* = 2.6 Hz, 3H, H-18), 0.65 (t, *J* = 4.4 Hz, 1H, H-4), 0.43 (dd, *J* = 8.0, 5.0 Hz, 1H, H-4). ^13^C NMR (101 MHz, CDCl_3_) *δ* 176.6 and 176.0 (C, C-26), 140.2 and 139.4 (CH, C-22), 128.6 and 127.9 (CH, C-23), 82. (CH, C-6), 60.2 and 60.0 (CH_2_, C-1′), 56.8 and 56.7 (CH, C-14), 56.0 (CH_3_, OCH_3_), 48.2 (CH, C-9), 48.1 (CH, C-17), 47.9 (CH, C-24), 44.6 and 44.4 (CH_3_, C-2′), 43.6 (C, C-10), 42.9 (C, C-13), 40.6 and 40.5 (CH, C-20), 40.3 (CH_2_, C-12), 35.4 (C, C-5), 35.2 (CH_2_, C-7), 33.5 (CH_2_, C-1), 30.6 (CH, C-8), 29.0 and 29.0 (CH_2_, C-15), 26.3 (CH_2_, C-11), 25.1 and 24.8 (CH_2_, C-2), 24.4 (CH_2_, C-16), 22.9 (CH_2_, C-28), 21.6 (CH, C-3), 21.2 and 21.1 (CH_3_, C-21), 19.4 (CH_3_, C-19), 14.8 (CH_3_, C-2′), 14.5 and 14.4 (CH_3_, C-27), 13.2 (CH_2_, C-4), 12.6 (CH_3_, C-18), 12.3 and 12.1 (CH_3_, C-29). HREIMS *m*/*z* 484.3911 (calcd for [C_32_H_52_O_3_]^•+^, 484.3910).

Ethyl (24*R*,25*RS*)-6β-Methoxy-3α,5-cyclo-5α-stigmastan-26-oate (**12**)

This compound was synthesized from Δ22 analogue **11** (0.45 g, 0.93 mmol) via catalytic hydrogenation using Pd on a charcoal catalyst, according to a procedure from the literature [26] as follows: yield: 0.40 g, 0.82 mmol, 88%. ^1^H NMR (500 MHz, CDCl_3_) *δ* 4.10–4.01 (m, 2H, H-1′), 3.25 (s, 3H, OCH_3_), 2.76–2.65 (m, 1H, H-6), 2.48–2.35 (m, 1H, H-25), 1.93–1.53 (m, 5H), 1.48–0.71 (m, 37H), 0.64 (d, *J* = 3.1 Hz, 3H, H-18), 0.60–0.56 (t, *J* = 4.4 Hz, 1H, H-4), 0.36 (dd, *J* = 8.0, 5.1 Hz, 1H, H-4). ^13^C NMR (126 MHz, CDCl_3_) *δ* 177.0 and 176.9 (C, C-26), 82.6 (CH, C-6), 60.1 (CH_2_, C-1′), 56.7 (CH_3_, OCH_3_), 56.67 and 56.65 (CH, C-14), 56.3 and 56.2 (CH, C-17), 48.2 (CH, C-9), 43.5 (C, C-10), 42.93 and 42.92 (C, C-13), 42.91 and 42.41 (CH, C-24), 41.7 and, 41.6 (CH, C-25), 40.43 and 40.40 (CH_2_, C-12), 36.4 and 36.2 (CH, C-20), 35.4 (C, C-5), 35.2 (CH_2_, C-1), 33.5 (CH_2_, C-7), 33.3 and 33.0 (CH_2_, C-22), 30.6 (CH, C-8), 28.5 and 28.4 (CH_2_, C-16), 27.3 (CH_2_, C-2), 26.2 (CH_2_, C-23), 25.1 (CH_2_, C-15), 24.4 and 24.3 (CH_2_, C-11), 23.0 and 22.9 (CH_2_, C-28), 21.7 (CH, C-3), 19.4 (CH_3_, C-19), 18.93 and 18.86 (CH_3_, C-21), 14.5 (CH_3_, C-2′), 13.2 and 13.0 (CH_2_, C-4), 12.5 and, 12.4 (CH_3_, C-27), 11.9 (CH_3_, C-18), 11.5 (CH_3_, C-29). HREIMS *m*/*z* 486.4068 (calcd for [C_32_H_54_O_3_]^•+^, 486.4064).

(24*R*,25*RS*)-6β-Methoxy-3α,5-cyclo-5α-stigmastan-26-ol (**13**)

To a stirred suspension of LiAlH_4_ (0.30 g, 8.0 mmol) in dry THF (30 mL) under nitrogen atmosphere, a solution of ethyl ester **12** (0.39 g, 0.80 mmol) in dry THF (20 mL) was added, and the mixture was stirred over night at room temperature. After the addition of the saturated aqueous NH_4_Cl solution (40 mL), the biphasic mixture was extracted with ethyl acetate (3 × 50 mL). The combined organic extracts were subsequently washed with water (2 × 100 mL) and brine (100 mL). After drying using a phase separation paper, the solvent was evaporated and the crude product was subjected to column chromatography (isohexanes/ethyl acetate 90:10) to provide (24*R*,25*RS*)-6β-methoxy-3α,5-cyclo-5α-stigmastan-26-ol (**13**) (0.34 g, 0.77 mmol, 96%) as a colorless oil. ^1^H NMR (500 MHz, CDCl_3_) *δ* 3.64–3.54 (m, 1H, H-26), 3.50–3.41 (m, 1H, H-26), 3.32 (s, 3H, OCH_3_), 2.77 (t, *J* = 2.9 Hz, 1H, H-6), 1.98 (dq, *J* = 12.5, 3.5 Hz, 1H, H-12), 1.89 (dt, *J* = 13.6, 3.2 Hz, 1H, H-7), 1.86–1.60 (m, 4H, H-8, H-25 and CH_2_), 1.51 (dt, *J* = 13.8, 7.9 Hz, 2H, CH_2_), 1.43–0.78 (m, 29H, with s, 3H, H-19 underneath), 0.71 (d, *J* = 1.9 Hz, 3H, H-18), 0.65 (t, *J* = 4.4 Hz, 1H, H-4), 0.43 (dd, *J* = 8.0, 5.1 Hz, 1H, H-4). ^13^C NMR (126 MHz, CDCl_3_) *δ* 82.6 (CH, C-6), 67.0 and 66.8 (CH_2_, C-26), 56.71 (CH_3_, OCH_3_), 56.67 and 56.66 (CH, C-14), 56.4 and 56.3 (CH, C-17), 48.2 (CH, C-9), 43.5 (C, C-7), 42.94 and 42.92 (C, C-13), 41.9 and 41.4 (CH, C-24), 40.5 and 40.4 (CH_2_,C-12), 37.51 and 37.49 (CH, C-25), 36.5 and 36.3 (CH, C-20), 35.5 (C, C-10), 35.2 (CH_2_, C-16), 34.2 and 33.9 (CH_2_, C-1), 33.5 (CH_2_, C-22), 30.6 (CH, C-8), 28.52 and 28.50 (CH_2_), 27.1 (CH_2_), 26.1 (CH_2_), 25.1 (CH_2_), 24.3 and 24.2 (CH_2_), 22.9 and 22.6 (CH_2_, C-11), 21.7 (CH, C-3), 19.4 (CH_3_, C-21), 19.0 and 18.9 (CH_3_, C-19), 13.2 (CH_2_, C-4), 13.1 (CH_3_, C-29), 12.54 and 12.45 (CH_3_, C-27), 12.4 (CH_3_, C-18). HREIMS *m*/*z* 444.3965 (calcd for [C_30_H_52_O_2_]^•+^, 444.3962).

(24*R*,25*RS*)-26-Ethynyl-6β-methoxy-26-nor-3α,5-cyclo-5α-stigmastane (**15**)

To a solution of (24*R*,25*RS*)-6β-methoxy-3α,5-cyclo-5α-stigmastan-26-ol (**13**) (0.33 g, 0.76 mmol) in dichloromethane (10 mL), PCC (0.33 g, 1.5 mmol) was added, and the mixture was stirred for 4 h at ambient temperature. The reaction mixture was filtered through a pad of celite, and the solvent was removed *in vacuo*. The residue was subjected to column chromatography (isohexanes/ethyl acetate 90:10), and the obtained crude product was used for the next step without further purification. To a cooled (0 °C) suspension of K_2_CO_3_ (0.090 g, 0.68 mmol) in methanol (5 mL), dimethyl (1-diazo-2-oxopropyl)phosphonate (Bestmann–Ohira reagent; 10% in acetonitrile, 1.2 mL, 0.51 mmol) was added under a nitrogen atmosphere, and the mixture was stirred for 5 min. The previously obtained crude aldehyde (0.15 g) was redissolved in dichloromethane (3 mL) and added dropwise. The mixture was allowed to warm to room temperature and stirred overnight. The saturated NH_4_Cl solution (1 mL) and water (8 mL) were added, and the biphasic mixture was extracted with dichloromethane (3 × 10 mL). The combined organic extracts were washed with brine (30 mL), and then dried using phase separation paper. After the solvent had been removed *in vacuo*, the crude product was subjected to column chromatography (isohexanes/ethyl acetate 98:2) to provide (24*R*,25*RS*)-26-ethynyl-6β-methoxy-26-nor-3α,5-cyclo-5α-stigmastane (**15**) (92 mg, 0.21 mmol, 28% over two steps) as a colorless oil. ^1^H NMR (400 MHz, CDCl_3_) *δ* 3.32 (s, 3H, OCH_3_), 2.77 (t, *J* = 2.9 Hz, 1H, H-6), 2.58 (td, *J* = 5.5, 2.4 Hz, 1H, H-25), 2.03–1.95 (m, 2H, H-12 and H-1′), 1.89 (dt, *J* = 13.7, 3.2 Hz, 1H, H-1), 1.86–1.55 (m, 4H), 1.53–1.04 (m, 20H), 1.02 (s, 3H, H-19), 0.96–0.75 (m, 10H), 0.72 (s, 3H, H-18), 0.67–0.62 (m, 1H, H-4), 0.43 (dd, *J* = 8.0, 5.0 Hz, 1H, H-4). ^13^C NMR (101 MHz, CDCl_3_) *δ* 88.7 and 88.3 (C, C-26), 82.6 (CH, C-6), 68.74 and 68.66 (CH, C-1′), 56.72 (CH_3_, OCH_3_), 56.7 (CH, C-14) 56.31 and 56.30 (CH, C-17), 48.2 (C-9), 44.9 and 44.8 (CH, C-24), 43.6 (C, C-19), 42.9 (C, C-13), 40.4 (CH_2_, C-12), 36.4 and 36.3 (CH, C-20), 35.5 (C, C-5), 35.2 (CH_2_, C-1), 33.8 (CH_2_, C-7), 33.5 and 33.4 (CH2, C-22), 30.6 (CH, C-8), 28.5 (CH_2_, C-16), 28.3 and 28.2 (CH, C-25), 27.3 (CH_2_, C-23), 26.6 (CH_2_, C-15), 25.1 (CH_2_, C-2), 24.4 and 24.3 (CH_2_, C-11), 23.3 and 22.9 (CH_2_, C-28), 21.7 (CH, C-3), 19.4 (CH_3_, C-19), 19.0 and 18.9 (CH_3_, C-21), 18.4 and 17.9 (CH_3_, C-27), 13.2 (CH_2_, C-4), 12.4 (CH_3_, C-18), 12.1 and 12.0 (CH_3_, C-29). HREIMS *m*/*z* 438.3856 (calcd for [C_31_H_50_O]^•+^, 438.3856).

(24*R*,25*RS*)-26-Ethynyl-26-norstigmast-5-ene-3β-ol, FB-DJ-1 (**4**)

(24*R*,25*RS*)-26-Ethynyl-6β-methoxy-26-nor-3α,5-cyclo-5α-stigmastane (**15**) (83 mg, 0.19 mmol) was dissolved in dioxane (3.5 mL) and water (0.50 mL), and *p*-toluenesulfonic acid (7.2 mg, 0.038 mmol) was added. The mixture was stirred at 80 °C for 30 min. After cooling to room temperature, the mixture was diluted with ethyl acetate (20 mL) and pyridine (0.75 mL) and washed with water (3 × 25 mL). After drying using phase separation paper, the solvent was evaporated, and the crude product was subjected to column chromatography (isohexanes/ethyl acetate 8:2) to provide (24*R*,25*RS*)-26-ethynyl-26-norstigmast-5-ene-3β-ol (**4**) (69 mg, 0.16 mmol, 86%) as a colorless solid. ^1^H NMR (500 MHz, CDCl_3_) *δ* 5.38–5.32 (m, 1H, H-6), 3.53 (dt, *J* = 14.9, 7.8 Hz, 1H, H-3), 2.58 (ddt, *J* = 7.0, 4.9, 2.5 Hz, 1H, H-25), 2.32–2.20 (m, 2H, H-4), 2.04–1.92 (m, 3H, H-7, H-12 and H-1′), 1.92–1.77 (m, 3H, H-1, H-2 and H-7), 1.54–0.88 (m, 30H, with s, 3H, H-19 underneath), 0.68 (s, 3H, H-18). ^13^C NMR (126 MHz, CDCl_3_) *δ* 140.9 (C, C-5), 121.9 (CH, C-6), 88.6 and 88.3 (C, C-26), 72.0 (CH, C-3), 68.8 and 68.7 (CH, C-1′), 56.9 (CH, C-14), 56.1 (CH, C-17), 50.3 (CH, C-9), 44.9 and 44.7 (CH, C-24), 42.48 (C, C-13), 42.46 (CH_2_, C-4), 39.9 (CH_2_, C-12), 37.4 (CH_2_, C-1), 36.7 (C, CH-10), 36.4 and 36.3 (CH, C-20), 33.8 and 33.4 (CH_2_, C-22), 32.1 (CH_2_, C-7), 31.8 (CH, C-8), 28.4 (CH_2_, C-2), 28.3 and 28.2 (CH, C-25), 27.3 (CH_2_, C-16), 26.5 (CH_2_, C-23), 24.5 and 24.3 (CH_2_, C-15), 23.3 (CH_2_, C-28), 21.2 (CH_2_, C-11), 19.6 (CH_3_, C-19), 19.0 and 18.9 (CH_3_, C-21), 18.3 and 17.9 (CH_3_, C-27), 12.1 (CH_3_, C-29), 12.01 and 11.98 (CH_3_, C-18). HREIMS *m*/*z* 424.3703 (calcd for [C_30_H_48_O]^•+^, 424.3700). m.p.: 116 °C. [α] = −25.8 ° (c = 0.16). Purity (HPLC): >99%.

## 3. Results

### 3.1. Synthesis Strategy

To synthesize the target compound FB-DJ-1 (**4**), there was a need for the construction of a Δ5-sterol with a side chain containing an ethyl branching at C-24 with exact and predictable 24*R* configuration, in combination with an alkyne moiety built up from one of the terminal methyl groups (C-26/C-27). This aim could not be achieved through a late-stage functionalization of sitosterol (**2**). The de novo enantioselective synthesis of complex steroids can only be achieved by means of complex multi-step procedures [27]. So, we intended to develop a synthetic approach which includes the assembly of the side chain at C-17 in a stereoselective manner, starting from an appropriately substituted steroidal precursor in the sense of a chiral pool synthesis [28,29], ideally utilizing asymmetric induction through existing stereocenters. Furthermore, the appropriate protection of the 3-OH and the Δ5 olefinic moiety had to be taken into consideration.

**Figure 2:**
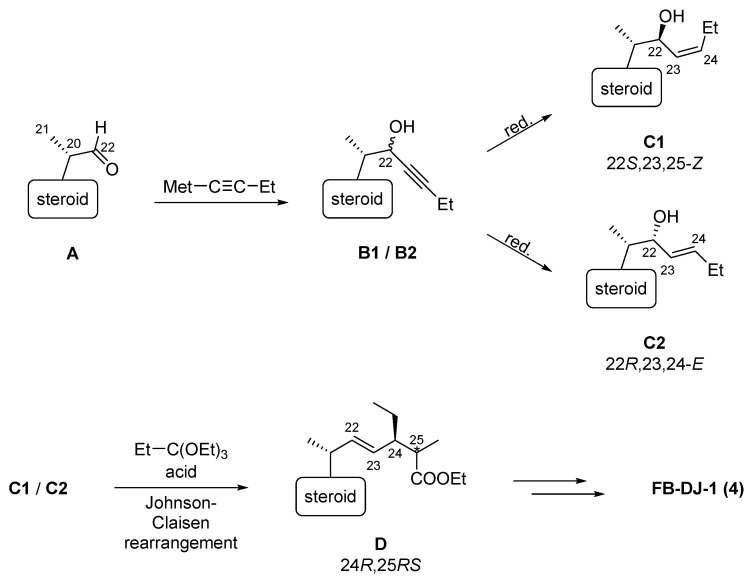
Envisaged synthesis route to the target compound FB-DJ-1 (**4**).

After the comprehensive analysis of published data in this field, we decided to utilize an approach, in which a crucial step is a Johnson–Claisen rearrangement, starting from an enantiopure steroidal side chain allylic alcohol and an orthocarboxylate, as described before by the groups of Djerassi [30] and Ikegawa [26], for the construction of the desired side chain. This procedure starts from a steroidal C-21 aldehyde (**A**), which is converted into a separable mixture of diastereomeric alkynols **B1/B2** by the addition of a metallated terminal alkyne (here, 1-butyne). The obtained alkynols can optionally be reduced to the (*E*)- or (*Z*)-configured allylic alcohols **C1/C2** via the use of a suitable reducing agent. From this point on, divergent routes should lead to branched side chains, including a terminal ester group, as potential precursors of the desired terminal alkyne. Notably, in this Johnson–Claisen rearrangement with triethyl orthopropionate, both a (22*R*)-configured alcohol with (*Z*)-geometry of the olefin (**C1**) and a (22*S*)-configured alcohol with (E)-geometry (**C2**) should provide products with identical (*R*)-configuration at C-24. Other than the configuration at C-24, the configuration at C-25 can not be fully controlled in this rearrangement; an epimeric mixture concerning C-25 was to be expected based on published data [28,29]. Finally, the ester group was to be converted into a terminal alkyne by means of reduction to the corresponding aldehyde, followed by alkyne synthesis utilizing the appropriate C_1_-building blocks (Figure 2).

### 3.2. Chemistry

**Scheme 1:**
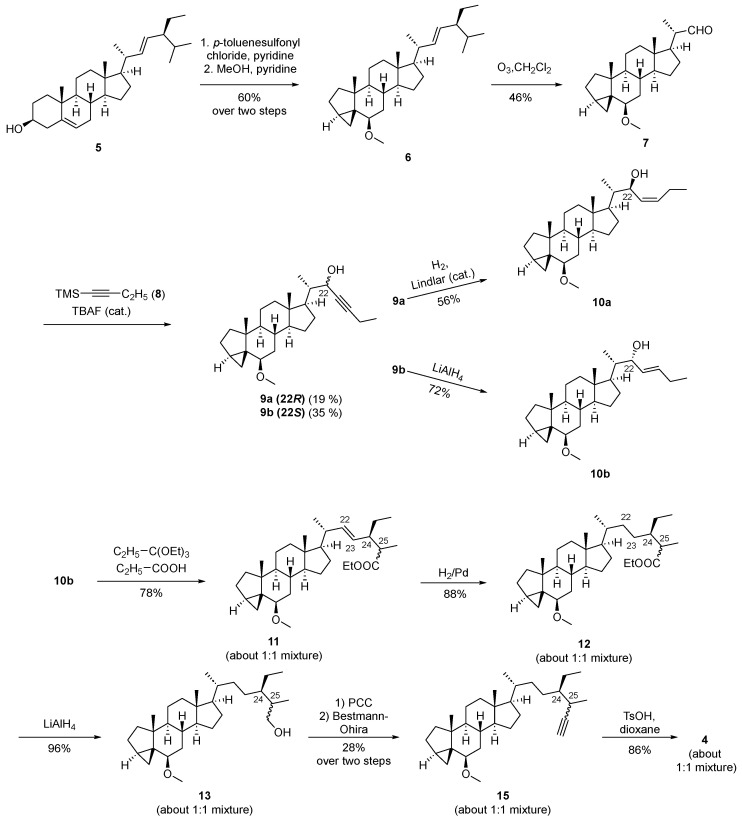
Synthesis of the target compound FB-DJ-1 (**4**).

As pointed out above, a suitably ring A/B-protected sterol with a truncated side chain (22-aldehyde) was required for this approach. We selected the commercially available sterol stigmasterol (**5**) as the starting material, since, after the appropriate protection of functional groups in the tetracyclic core, the Δ22 double bond can be cleaved via ozonolysis to give the required aldehyde building block **7**. The protection of the 3-hydroxy group and the Δ5 olefinic moiety had to be resistant to all of the intended modifications in the side chain, including ozonolysis, organometallic chemistry, reductions, acid-mediated Johnson–Claisen rearrangement, and aldehyde-to-alkyne conversion. For this purpose, we identified the *i*-methyl ether **6** as the most promising intermediate, since it includes the protection of both the 3-OH and the Δ5 olefinic moiety and shows high stability, but also allows for the easy regeneration of intact ring A/B functionalities via treatment with acids. Intermediate **6** was obtained from stigmasterol (**5**) following a published protocol, and the subsequent ozonolysis of the Δ22 olefin produced aldehyde **7** in an acceptable yield [31] (Scheme 1).

In the next step, 1-metalated 1-butyne was to be added to the aldehyde **7** in order to obtain the alkynol intermediates of type **B1/B2** (Figure 1). In previous works in this field, this nucleophilic addition was performed by using 1-butyne (boiling point: 8 °C) after the conversion of the terminal alkyne moiety into the corresponding bromomagnesium [26,30] or lithium salts [25]. But, since 1-butyne is commercially available only in a gaseous form in steel bottles, and the handling of this compound bears severe risks due to its explosiveness [32], we intended to avoid using this hazardous compound. So, we chose to utilize 1-trimethylsilyl-1-butyne (**8**), a commercially available, easy-to-handle liquid, as the precursor for a 1-metalated 1-butyne. A limited number of publications describe in situ generations of acetylides from 1-trimethylsilylalkynes and fluoride sources, followed by additions to aldehydes or ketones. In situ desilylation was accomplished by means of fluoride sources, like tetrabutylammonium fluoride (TBAF) [33,34] and cesium fluoride [35], the latter in some cases promoted by the addition of a crown ether [36,37]. In fact, aldehyde **7,** upon treatment with 1-trimethylsilyl-1-butyne (**8**) and catalytic amounts of TBAF, provided a diastereomeric mixture of alkynols (**9a/9b**), which was, as published [25,26,30], separable on a preparative scale via the use of flash column chromatography on silica gel. The best yields (35% for the 22*S*-enantioner **9b**, 19% for the 22*R*-enantiomer **9a**) were obtained via four equivalents of the alkyne building block and very slow addition to the aldehyde solution. These yields are in the same range as obtained by means of the addition of 1-butyne building blocks, obtained via the direct metalation of the alkyne [25,30].

As pointed out above, both diastereomers (**9a** with 22*R* configuration and **9b** with 22*S* configuration) should open the option for synthesizing the 24*R*-configured target compound by means of a Johnson–Claisen rearrangement. For this purpose, **9a** (22*R*) needed to be reduced in a stereoselective manner to the *Z*-olefin **10a**, whereas **9b** (22*S*) was to be converted into the *E*-olefin **10b**. Both of these reductions were conveniently achieved following published protocols as follows: *Z*-configured allylic alcohol **10a** was obtained (56% yield) via the reduction of **9a** by means of hydrogenation with Lindlar’s catalyst [31], and *E*-configured product **10b** was obtained in 72% yield via the lithium alanate reduction of **9b** [28].

The construction of C24-branched sterols from side chain allylic alcohols has been the subject of previous investigations, and the hydroxy group at C-22 offers, via derivatization with activated carboxylic acids, a central step. The previous work of Horibe et al. [38] included esterification and subsequent transformation into ester silylenol derivatives, followed by the rearrangement. However, this reaction affords the cancerogenic solvent HMPA, and thus is not practicable nowadays. A convenient alternative was formulated by Djerassi [28], who exploited the direct conversion of allylic alcohols with orthocaboxylates. Following this and other related protocols [26] with some modifications, alcohols **10a/10b** were treated with triethyl orthopropinate/propionic acid in refluxing xylene.

To our surprise, and in contrast to published data [26,38], the *Z*-configured alcohol **10a**, upon treatment with triethyl orthopropionate, provided a complex mixture of stereoisomeric products (evident from additional resonances in the NMR spectra when compared to the sample obtained from **10b**), from which the target product could not be isolated. Fortunately, the *E*-configured alcohol **10b** reacted readily with triethyl orthopropionate/propionic acid in the manner of a Johnson–Claisen rearrangement to give ester **11** in a 78% yield. As expected from the published data, we obtained a mixture of epimers, since no stereocontrol was obtained for C-25. The epimeric esters **11** were not separable on a preparative scale, and represent (based on ^13^C-NMR data; see Supplementary Materials) about a 1:1 mixture.

The reduction of the Δ22 double bond in **11** to give ester **12** was easily achieved via catalytic hydrogenation using Pd on the charcoal catalyst [26], providing saturated derivative **12** in a 88% yield. In the final and unprecedented step, the ester group of **12** had to be converted into a terminal alkyne. This was accomplished in three steps, starting with the reduction of the ester moiety to a primary alcohol **13** with lithium alanate (96% yield). The subsequent controlled oxidation of **13** to the aldehyde **14** was first attempted with Dess–Martin periodinane [39], but resulted in a disappointing yield (24%). In contrast, oxidation with PCC (pyridinium chlorochromate) [40] led to clean and complete conversion within 4 h; however, aldehyde **14** turned out to decompose rapidly. Consequently, we only performed a very short chromatographic preliminary purification, and subjected the obtained product directly to a Bestmann–Ohira reaction [41] in order to obtain the alkyne **15** in a 28% yield over two steps. In the final step, both the 3-OH and the Δ5 moieties were regenerated from the steroid *i*-methyl ether in an 86% yield via the treatment with *p*-toluenesulfonic acid in dioxane. The obtained target compound FB-DJ-1 (**4**) was, as expected, about a 1:1 mixture of epimers at C-25; neither this compound nor the precursors (**12**, **13**, **15**) were separable via chromatography on a preparative scale (Scheme 1).

## 4. Discussion

For an in-depth investigation of the localization and biological functions of phytosterols, there is an urgent need for phytosterol-based chemical tools, which show, on the one hand, close structural similarity to the physiological sterols, but, on the other hand, offer options for derivatization with fluorescent dyes by means of click chemistry for highly sensitive fluorescence imaging. Based on published methods utilizing the Johnson–Claisen rearrangement for the construction of branched sterol side chains, and with major novel methodologic input, we formulated the synthesis of the promising alkyne-derived sitosterol FB-DJ-1 (**4**) for future investigations of plants. This new chemical probe is at present under investigation (in combination with novel azide dyes) in the lab of our cooperative partners for imaging experiments in diverse plants. 

Moreover, the synthesis protocol described here offers the opportunity for preparing related interesting phytosterol-derived chemical probes. For instance, maintaining the Δ22 double bond (see intermediate 11) would lead to clickable alkyne derivatives of stigmasterol (5), and, with slight modifications, alkyne derivatives of 24*S*-configured algal sterols (e.g., clionasterol (3)) should be available. Furthermore, the 26-hydroxy intermediate 13 could alternatively be converted into an azide, providing complementary clickable chemical tools, as compared to the alkyne **4** and analogues discussed before.

Thus, we are confident that the chemical approach we present here will open doors for future comprehensive imaging experiments in the field in phytosterols.

## Data Availability

Detailed data are available upon request from the corresponding author.

## Supplementary Materials

The following supporting information can be downloaded at https://www.mdpi.com/article/10.3390/biom14050542/s1, NMR spectra of all synthesized compounds.
