# Supplementary material for: Synthesis of a Side Chain Alkyne Analogue of Sitosterol as a Chemical Probe for Imaging in Plant Cells
Source: Biomolecules. 2024 Apr 30;14(5):542. doi: 10.3390/biom14050542 (PMC11117550; doi:10.3390/biom14050542)

Synthesis of a Side Chain Alkyne Analogue of Sitosterol as a Chemical Probe for Imaging in Plant Cells

MMiriam Hollweck, David Jordan and Franz Bracher *

Supporting Information

^{1}H and ^{13}C NMR spectra of the synthesized compounds

(24*R*,25*RS*)-26-Ethynyl-26-norstigmast-5-ene-3β-ol, FB-DJ-1 (**4**)

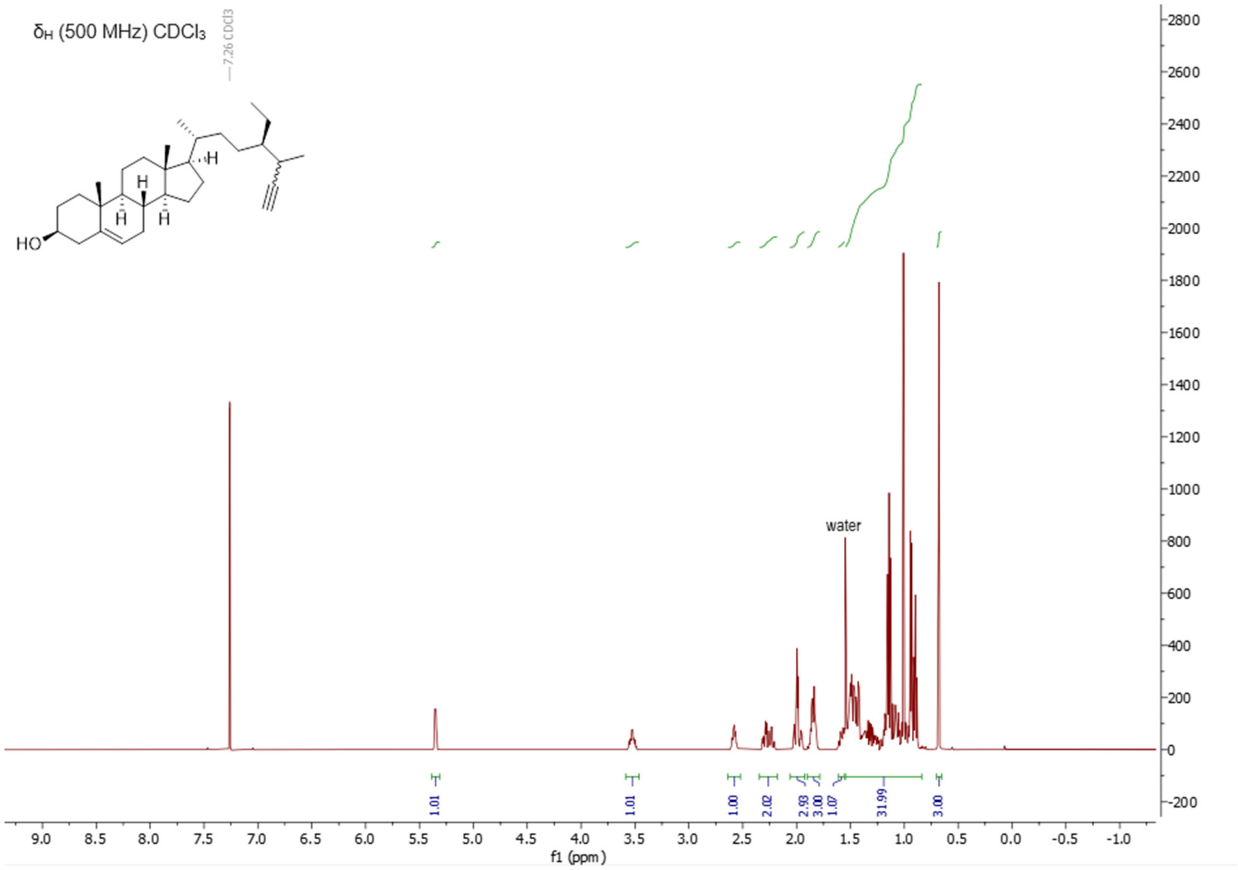

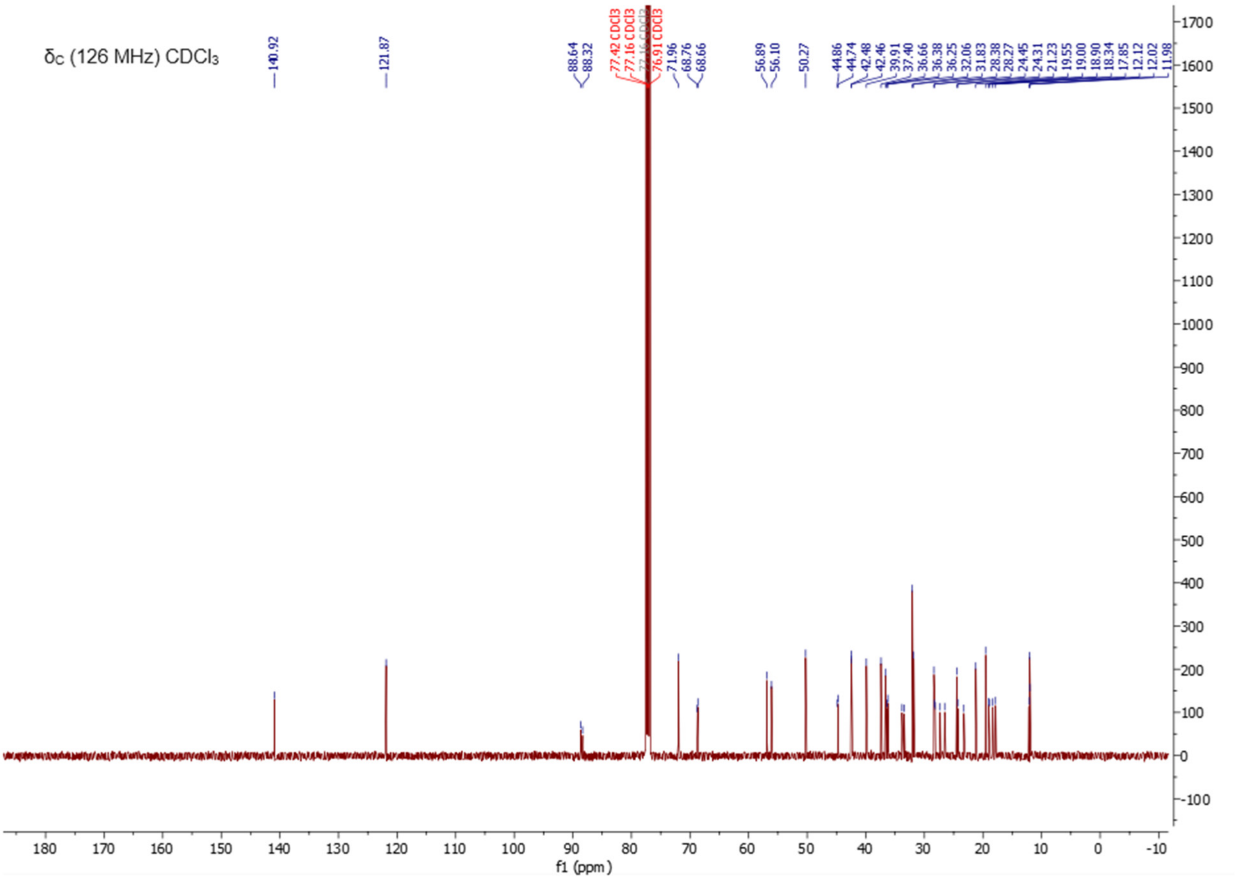

(22*R*)-6β-Methoxy-3α,5-cyclo-27-nor-5α-cholest-23-yn-22-ol (**9a**)

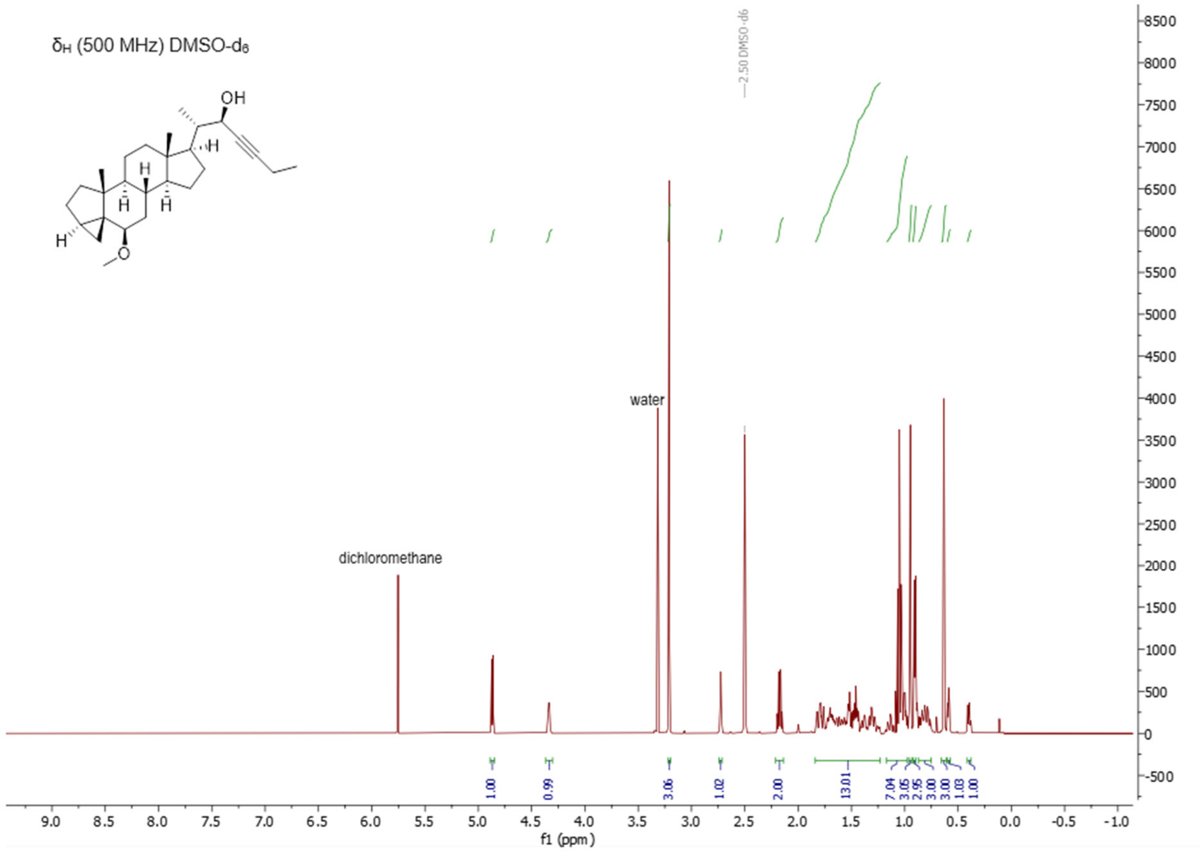

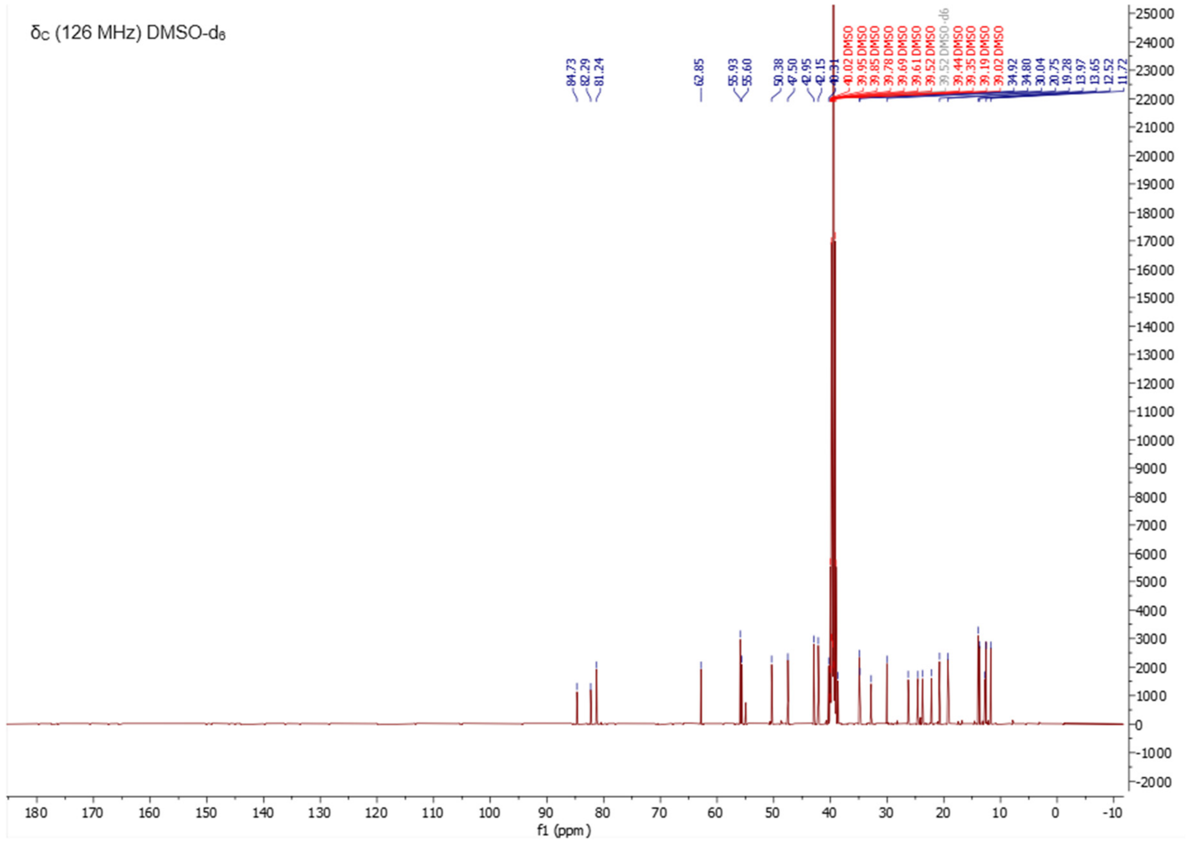

(22*S*)-6β-Methoxy-3α,5-cyclo-27-nor-5α-cholest-23-yn-22-ol (**9b**)

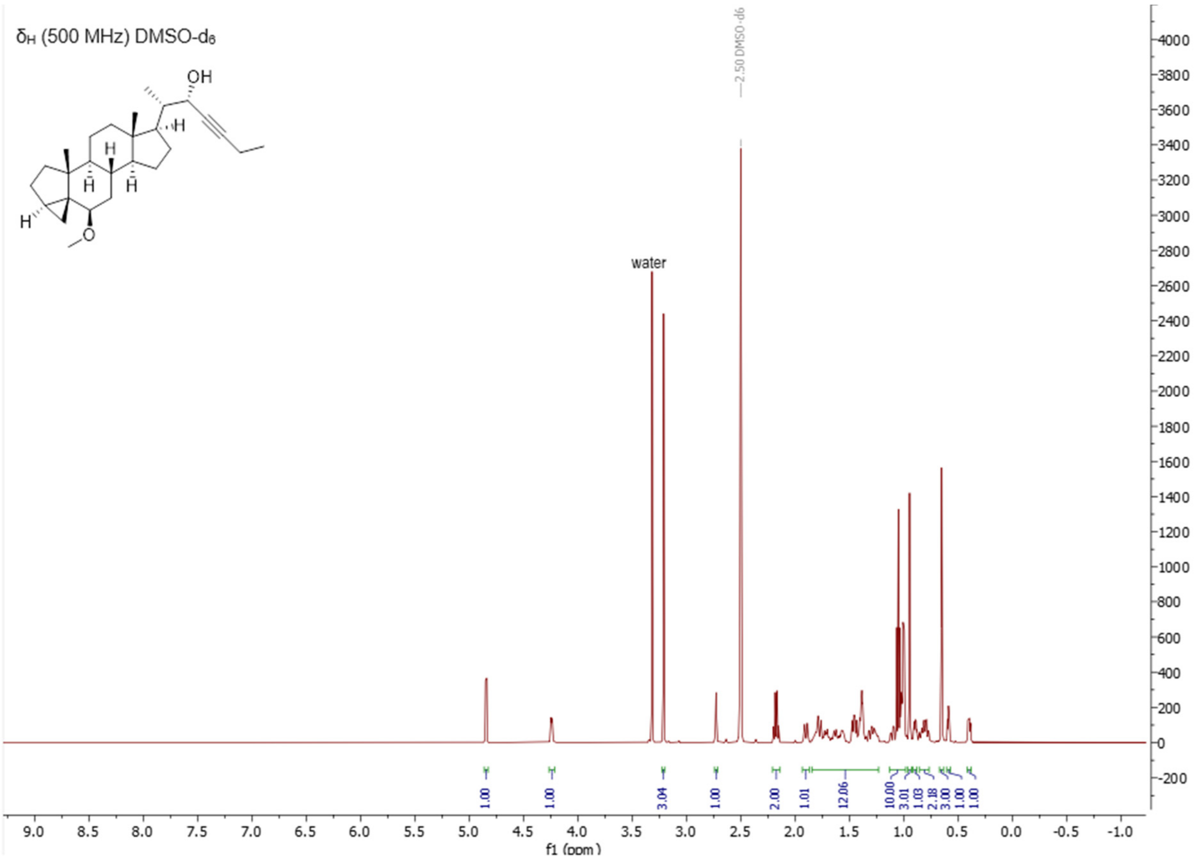

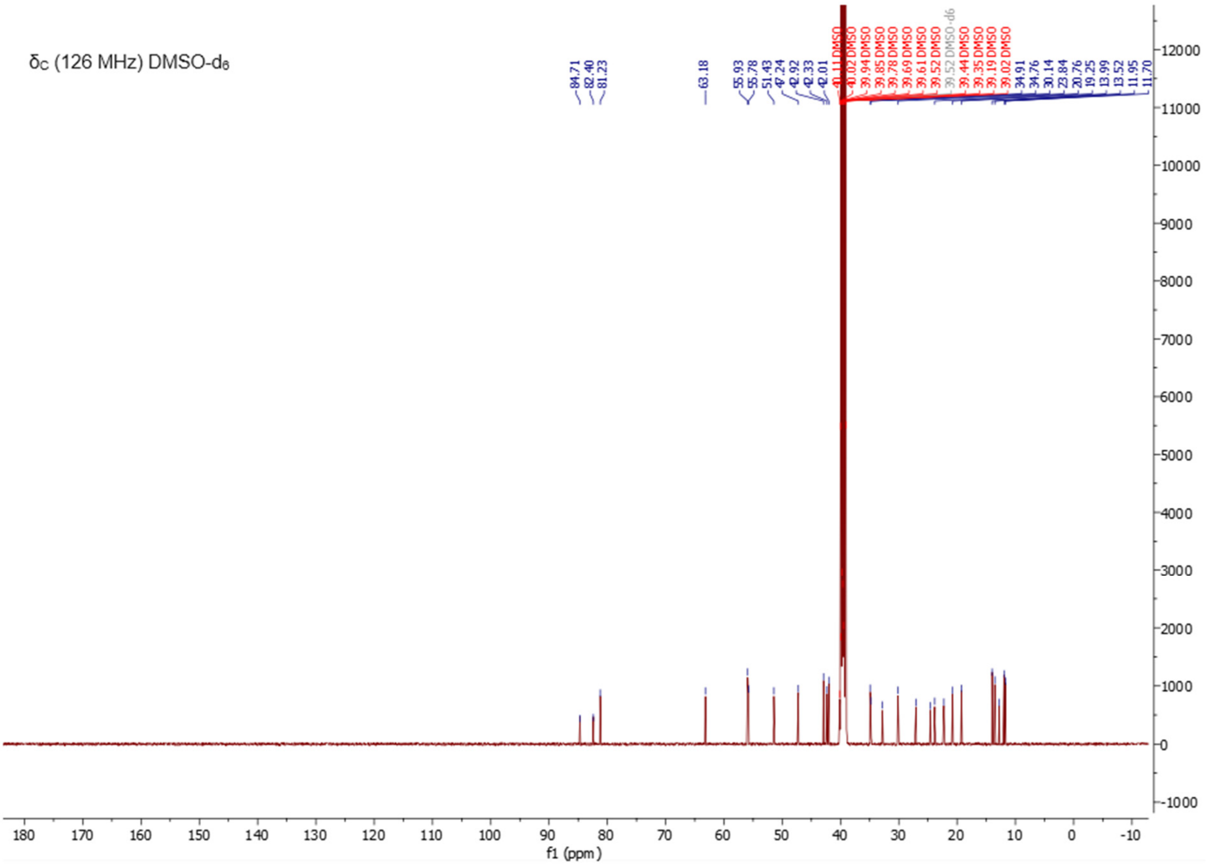

(22*R*,23*Z*)-6β-Methoxy-3α,5-cyclo-27-nor-5α-cholest-23-en-22-ol (**10a**)

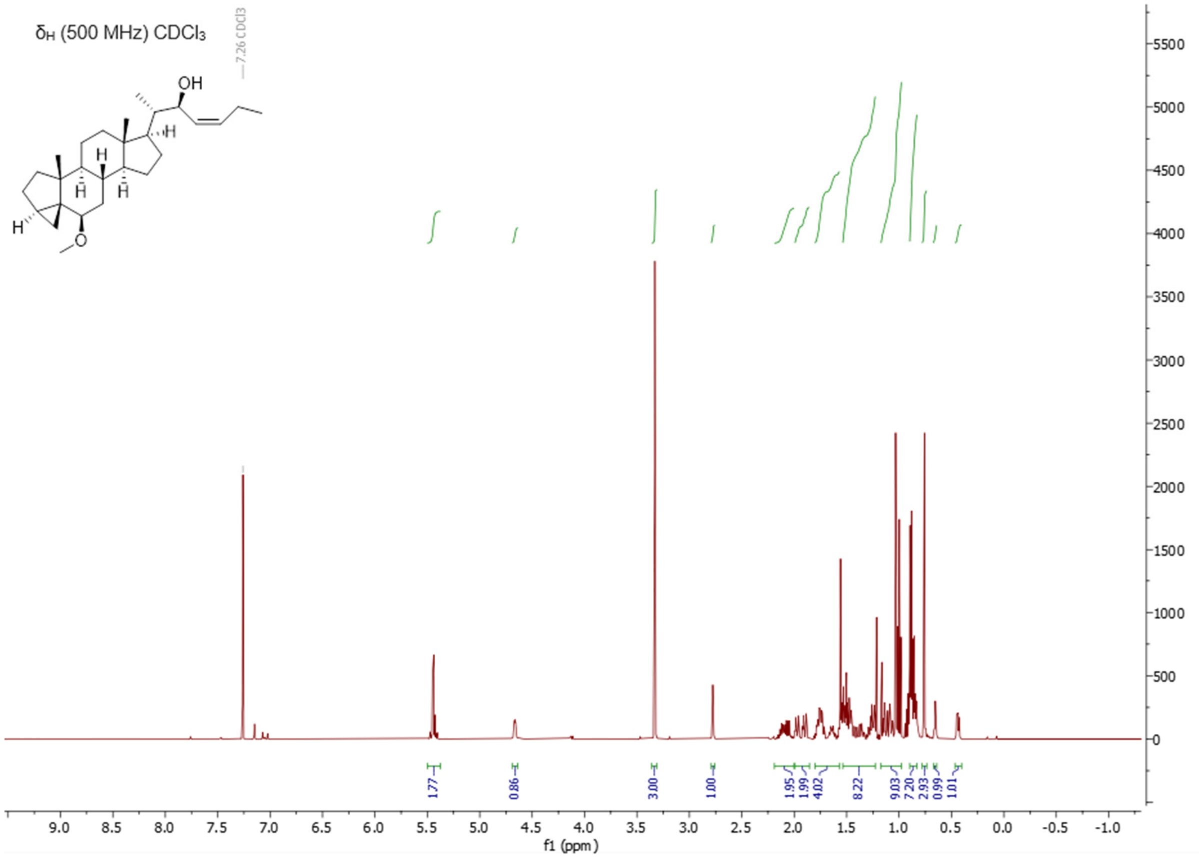

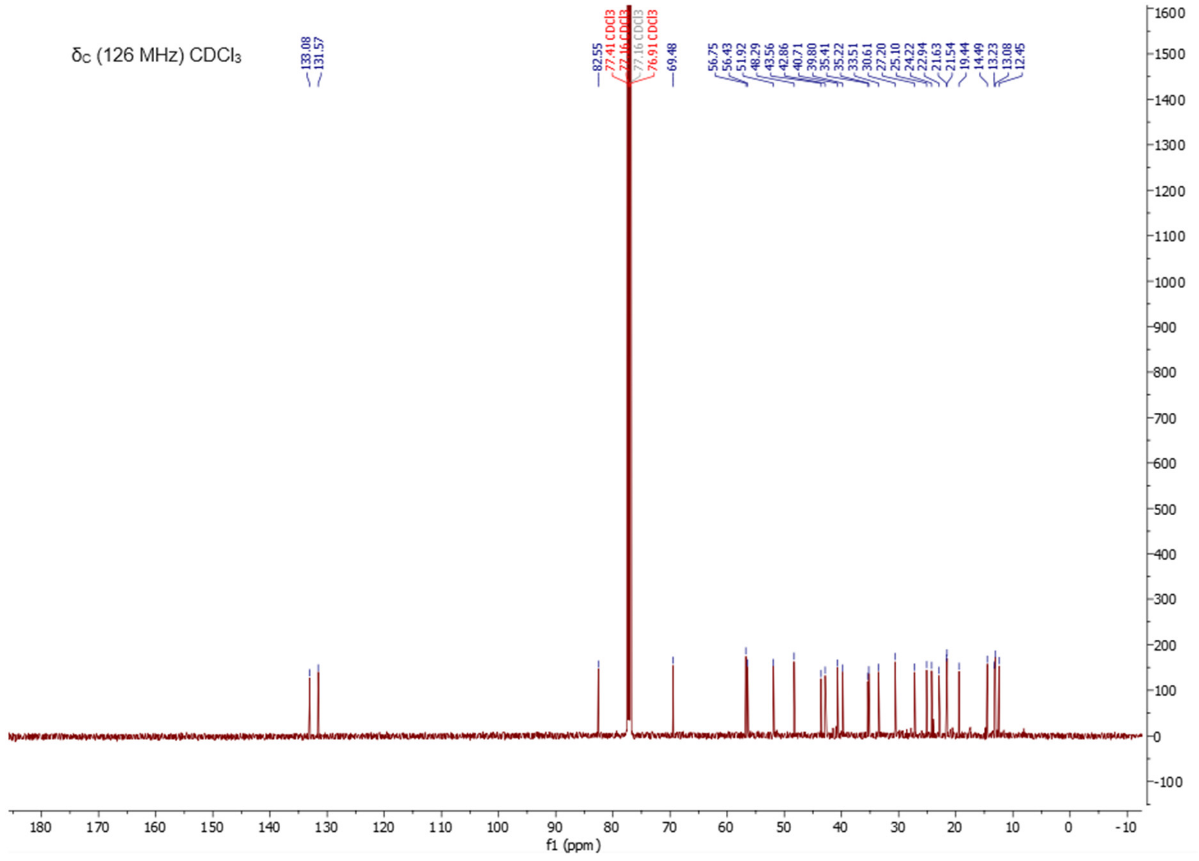

(22*R*,23*E*)-6β-Methoxy-3α,5-cyclo-27-nor-5α-cholest-23-en-22-ol (**10b**)

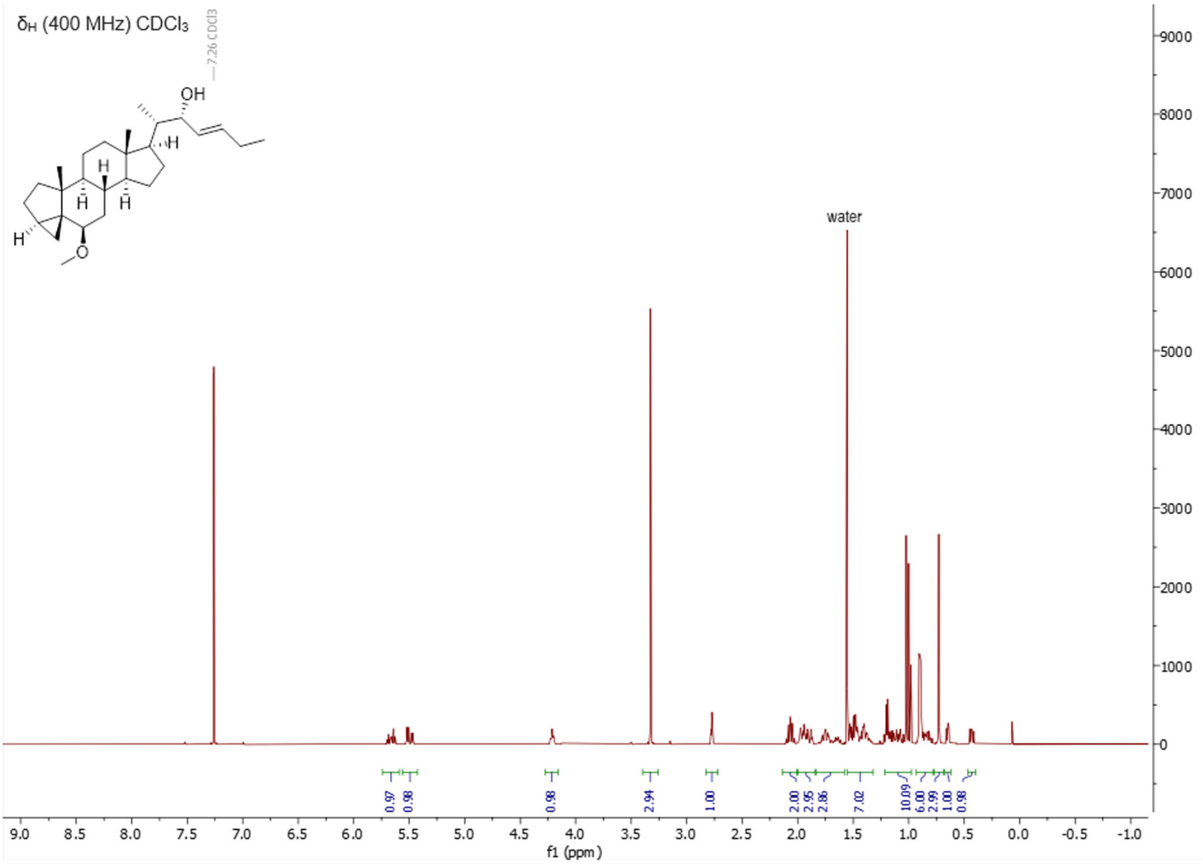

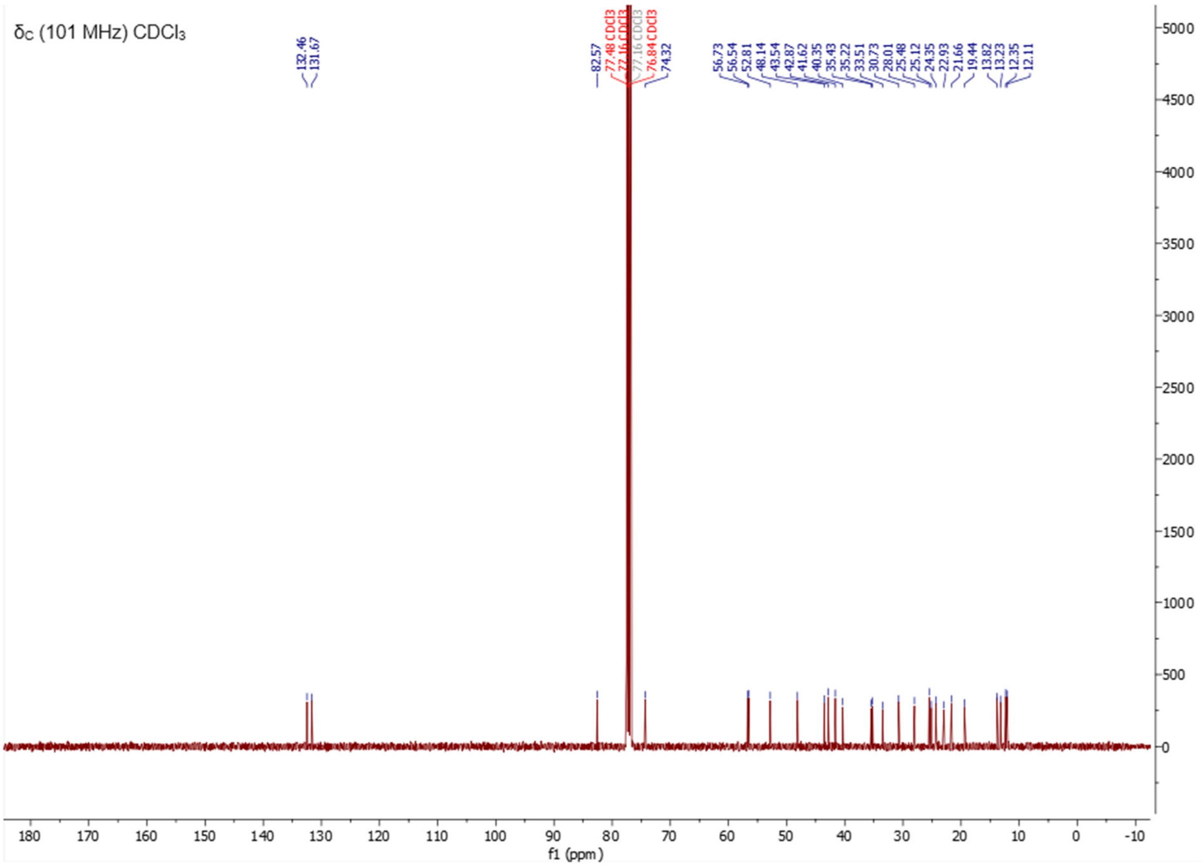

Ethyl-(22*E*,24*R*,25*RS*)-6β-Methoxy-3α,5-cyclo-5α-stigmast-22-en-26-oate (**11**)

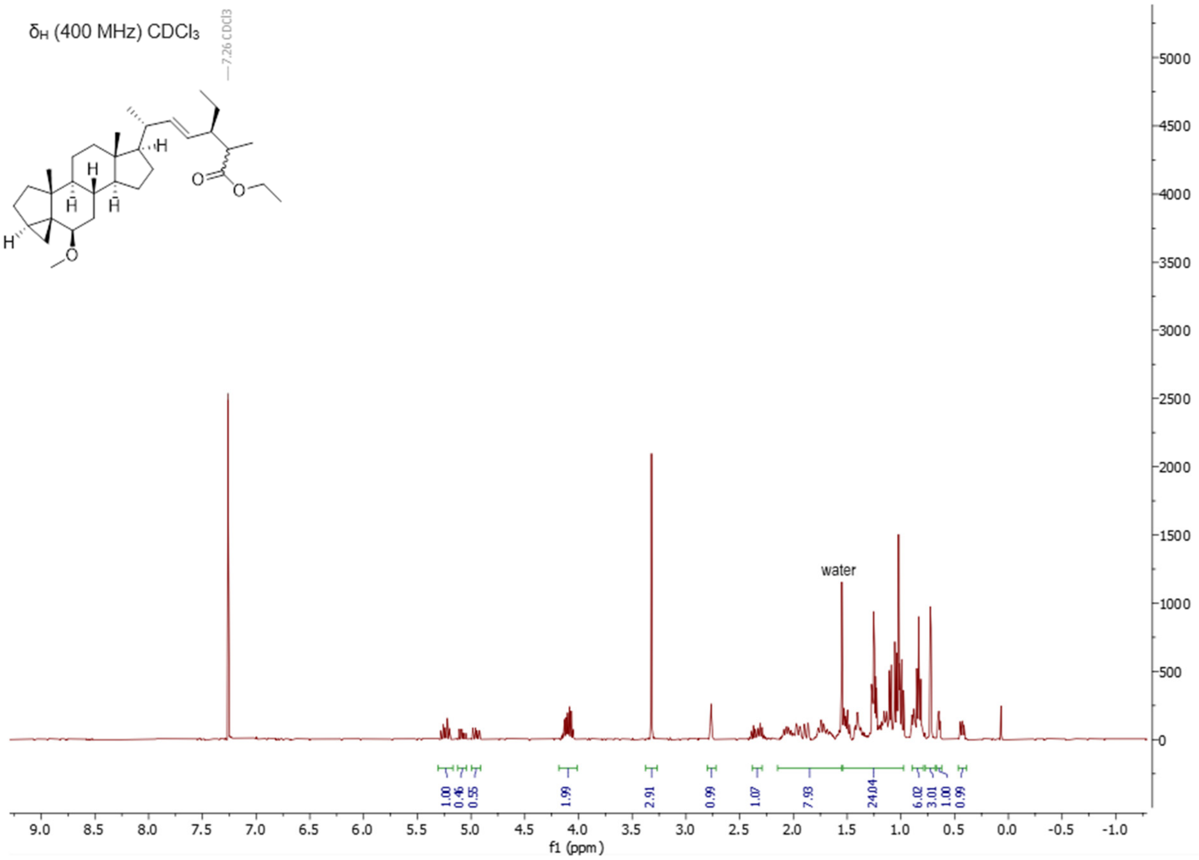

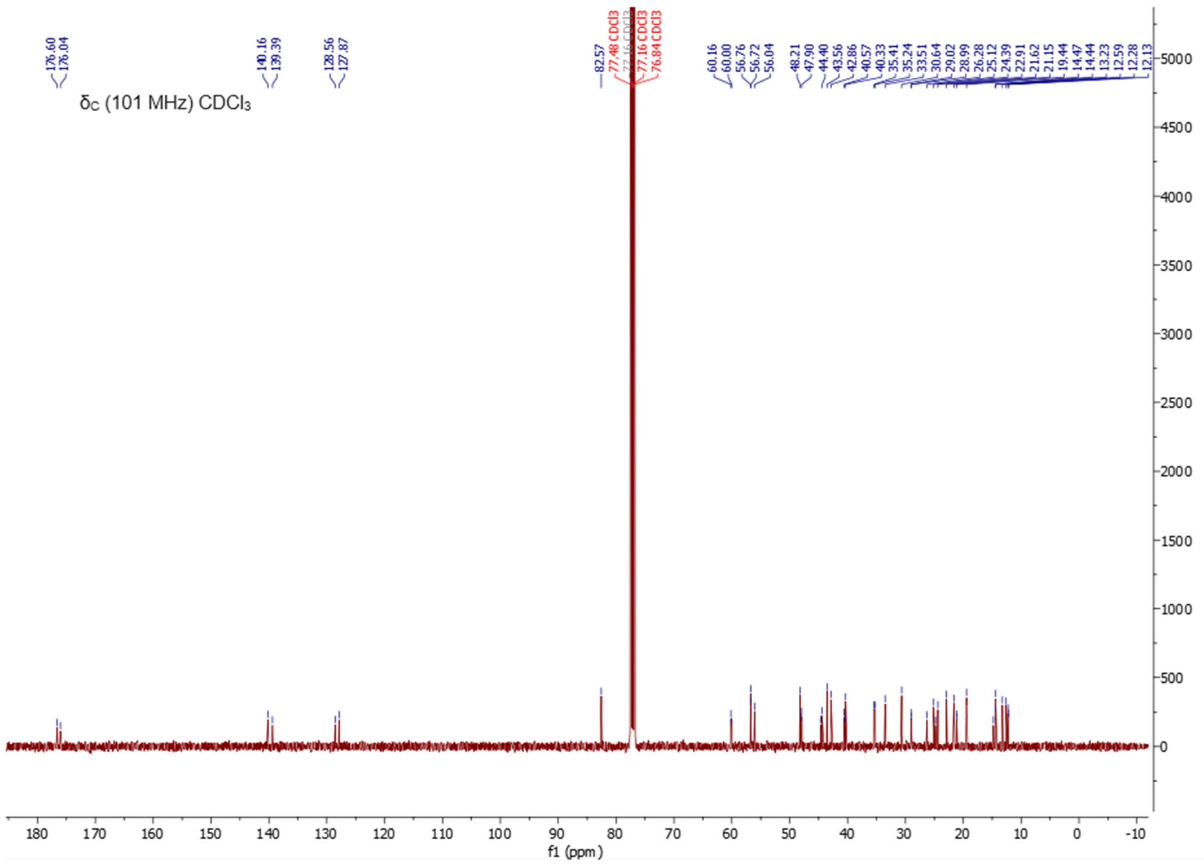

Ethyl-(24*R*,25*RS*)-6β-Methoxy-3α,5-cyclo-5α-stigmastan-26-oate (**12**)

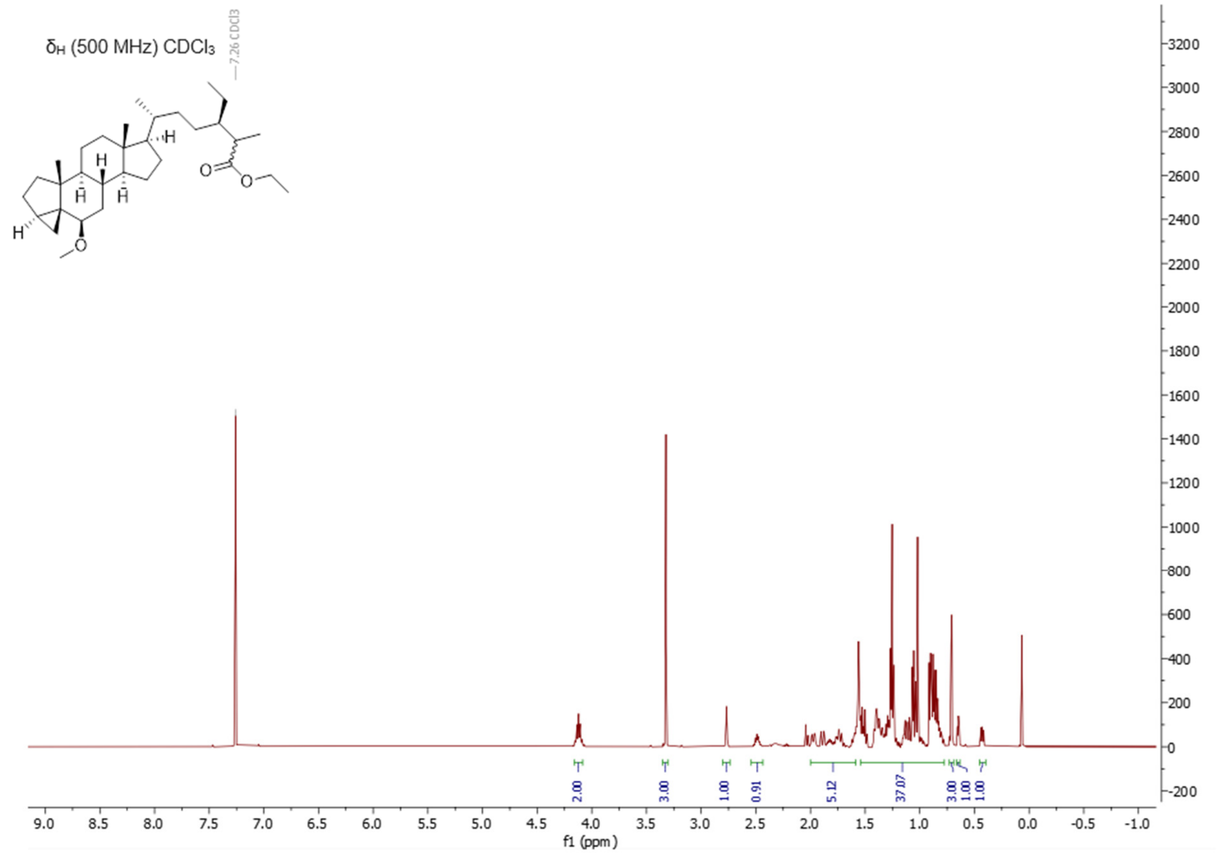

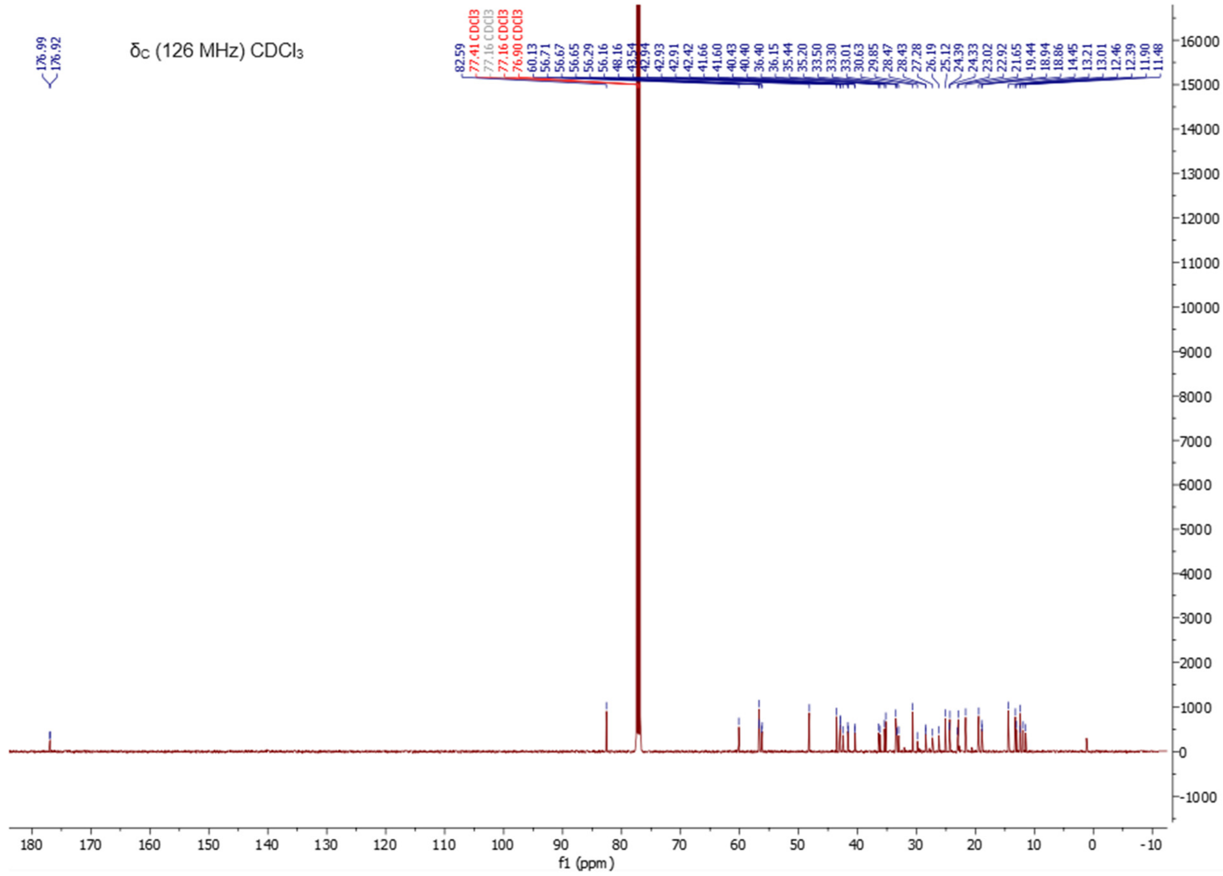

(24*R*,25*RS*)-6β-Methoxy-3α,5-cyclo-5α-stigmastan-26-ol (**13**)

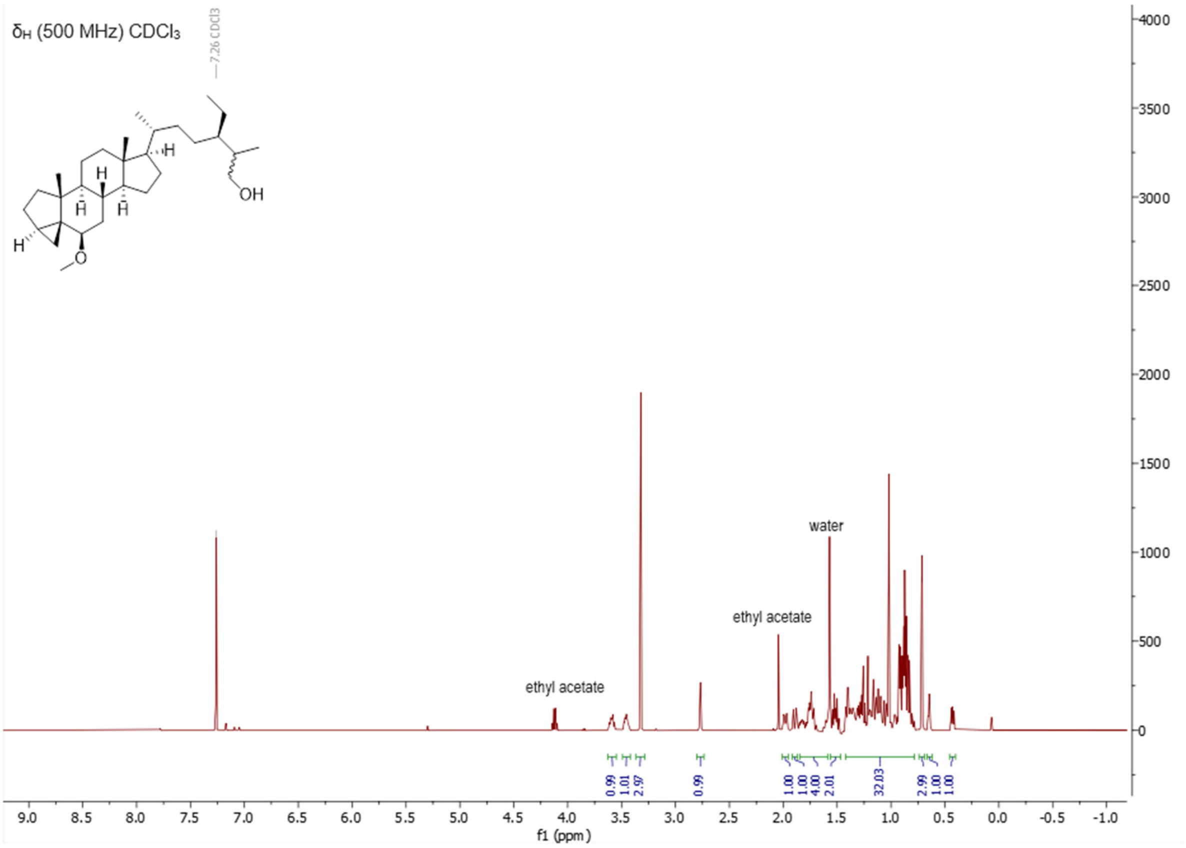

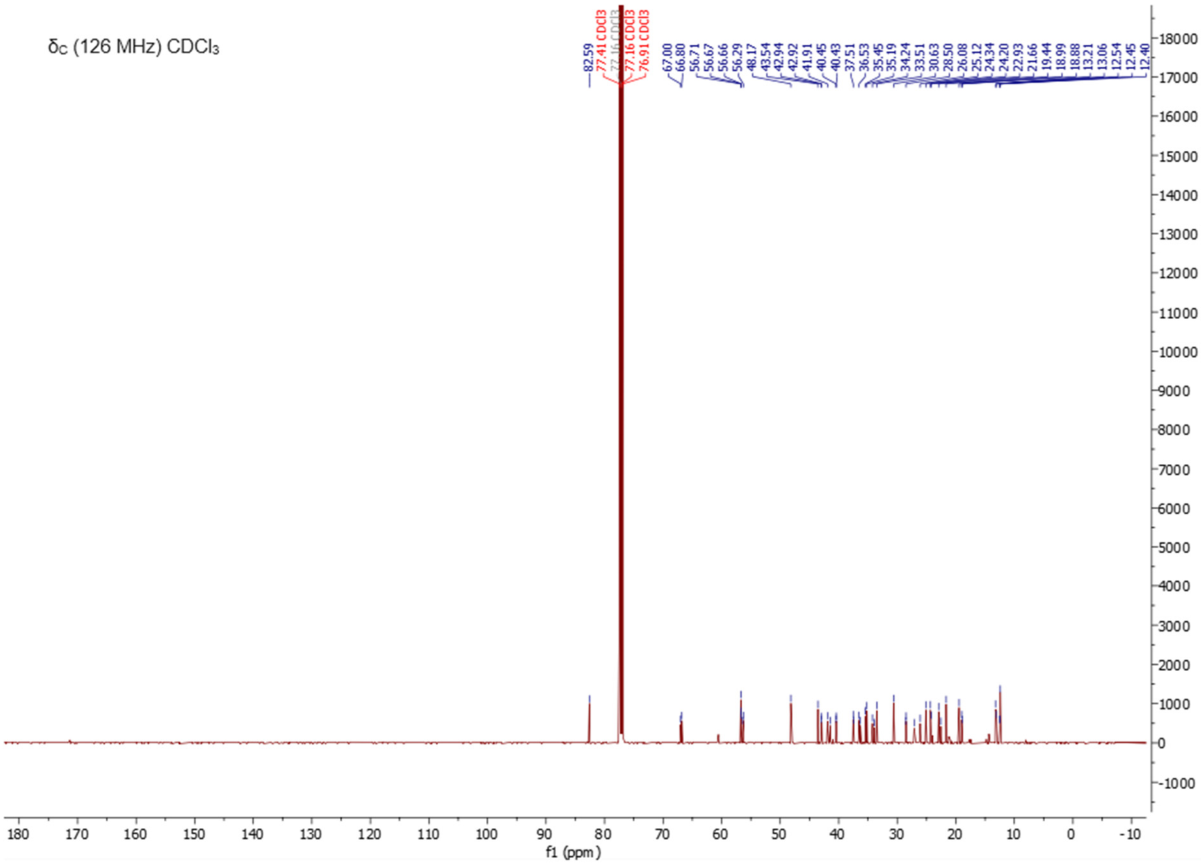

(24*R*,25*RS*)-26-Ethynyl-6β-methoxy-26-nor-3α,5-cyclo-5α-stigmastane (**15**)

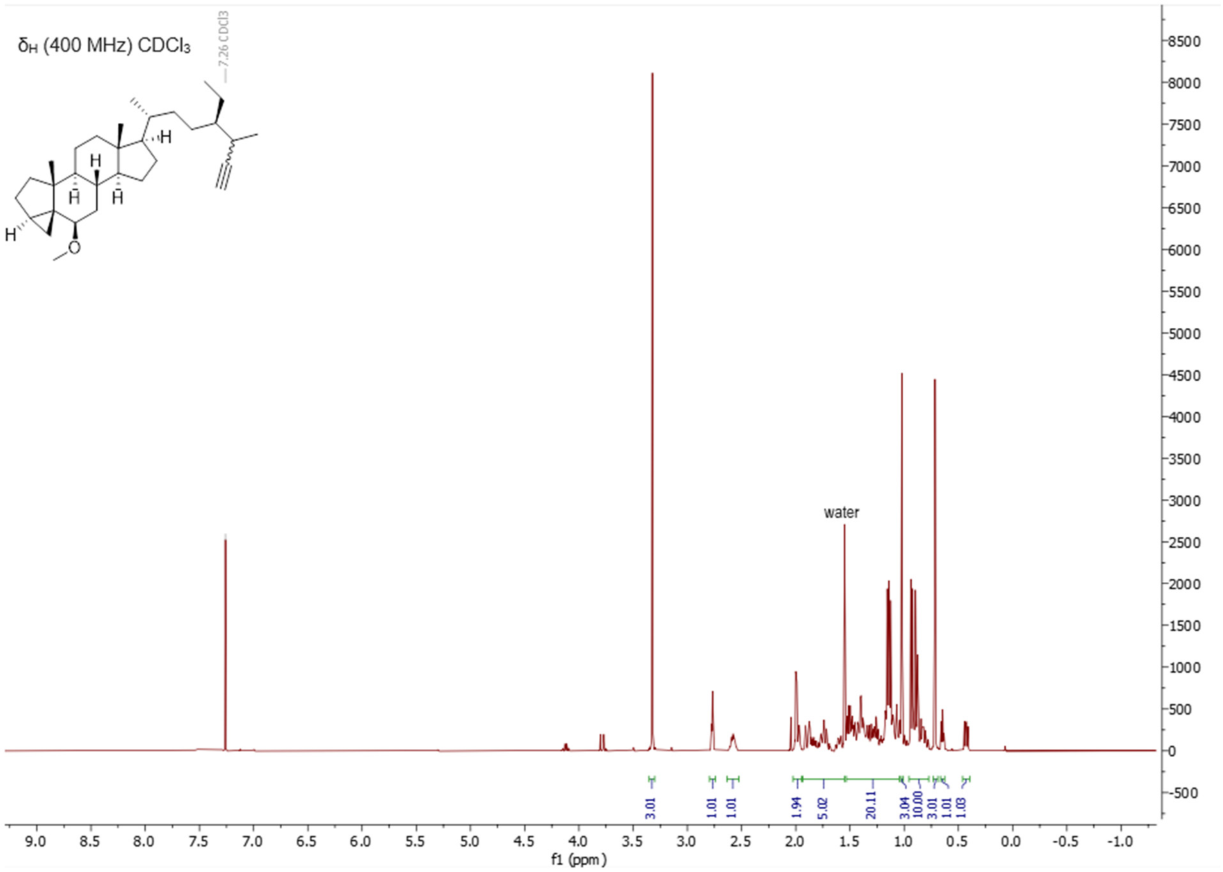

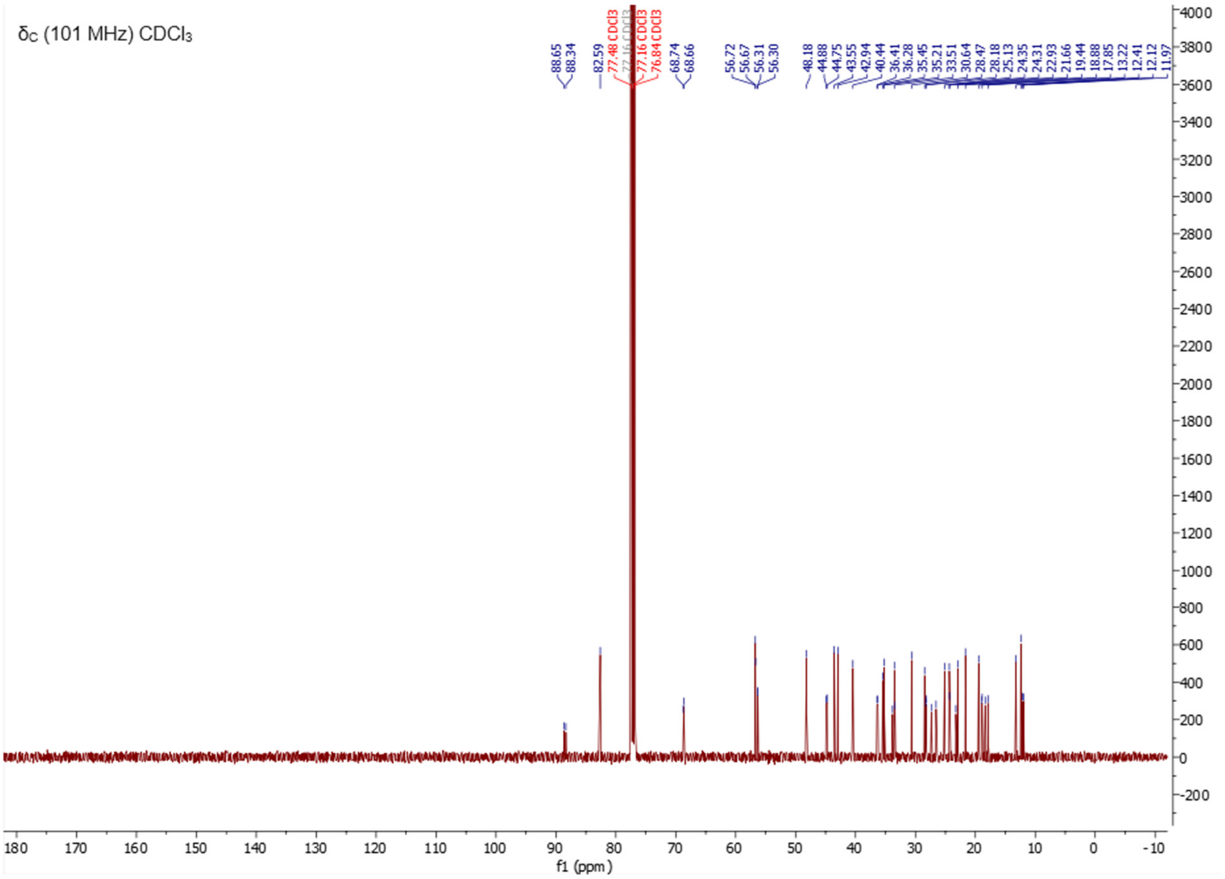

Supplement: Supplementary file 1 [file biomolecules-14-00542-s001.zip › biomolecules-2984733-supplementary.pdf]
